# The effect of black tea and water temperature on the disintegration of gelatine and HPMC capsules, tested with the paddle device, GastroDuo and *in vivo* pharmacokinetics: Much ado about little

**DOI:** 10.1016/j.ijpx.2025.100342

**Published:** 2025-05-27

**Authors:** Dorota Sarwinska, Mathilde Leyh, Constantin Foja, Theodora Tzakri, Philipp Schick, Felix Morof, Julius Krause, James Mann, Richard Barker, Mladen Vassilev Tzvetkov, Werner Weitschies, Michael Grimm

**Affiliations:** a Center of Drug Absorption and Transport, Department of Biopharmaceutics and Pharmaceutical Technology, Institute of Pharmacy, University of Greifswald, Felix-Hausdorff-Str. 3, 17489 Greifswald, Germany; b Department of Clinical Pharmacology, University Medicine Greifswald, 17487 Greifswald, Germany; c Oral Product Development, Pharmaceutical Technology & Development, Operations, AstraZeneca, Macclesfield, UK; d New Modalities & Parenteral Development, Pharmaceutical Technology & Development, Operations, AstraZeneca, Macclesfield, UK

**Keywords:** Capsules, Pharmacokinetics, Black tea, Temperature effect, USP 2, GastroDuo, Salivary tracer technique

## Abstract

Fluid co-administered with oral medication directly affects its behaviour. Often, people use fluids other than water when taking their medications. Capsules (mainly gelatine- and HPMC-based) are widely used solid oral dosage forms. The presented study aimed to investigate the behaviour of gelatine and HPMC capsules in several fluids *in vitro* and *in vivo*. The second aim was to assess the influence of administered fluids on the gastric emptying. The third aim was to assess the usability and predictive power of different *in vitro* methods for drug analysis and compare them with *in vivo* data. For *in vitro* studies, two systems with different complexities were used: the compendial USP 2 apparatus and the GastroDuo biorelevant model. In both systems, 25 mL of SGF and 240 mL of tested fluid were used. To obtain the *in vivo* data, a clinical study with 12 young and healthy volunteers was performed. In this study, the salivary tracer technique, which utilises caffeine kinetics as a marker of a dosage form behaviour in the GIT, was used. *In vitro*, the temperature strongly affected the opening times of gelatine capsules (rapid opening in warm media and slower in cold). *In vivo*, the opening time of gelatine capsules in warm black tea was slightly delayed in comparison to warm water. The differences in opening times between warm and cold water and warm black tea and cold water were significant. In USP 2 Apparatus, HPMC capsules were more sensitive to the tested media than in the biorelevant GastroDuo model. There were no significant differences in the opening times of HPMC capsules *in vivo*. Gastric emptying of warm water, cold water or warm black tea was not affected, suggesting that the altered *in vivo* absorption kinetics was caused by the *in vivo* behaviour of the capsules, depending on their properties and not by changes in the gastric emptying of the co-administered fluids. The presented study allows a better understanding of gelatine and HPMC capsules behaviour *in vitro* and *in vivo* administered with different fluids. Moreover, it demonstrated the relevance of *in vivo* data as well as the limitations of *in vitro* tools.

## Highlights

• GastroDuo demonstrated better predictive power than the USP 2 apparatus.
• *In vitro* real-life fluids influenced capsules behaviour more than *in vivo*.
• HPMC capsules were not influenced by temperature or black tea *in vivo*.
• Temperature affected the opening times of gelatine capsules *in vivo*.
• Warm and cold water and warm black tea did not influence gastric emptying.

## Graphical Abstract

**Figure:**
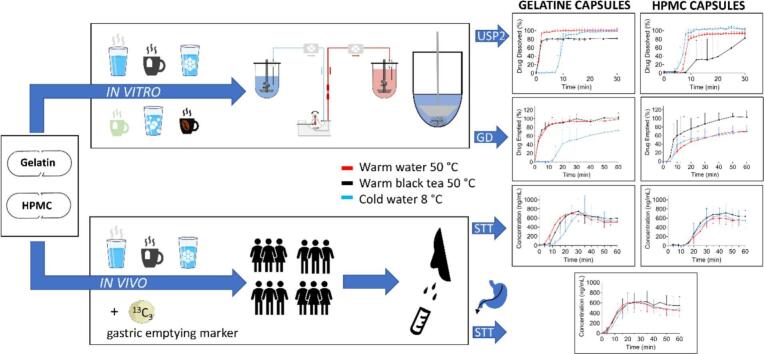

## 1 Introduction

The oral route is the most common and often preferred way of drug administration, due to its convenience in administration and handling (Debotton and Dahan, 2017). For most patients, this route is the most comfortable and the least problematic. However, from a biopharmaceutical perspective, the oral route of administration is also challenging. First, because of the acidic environment of the stomach (pH between 1 and 2.5), some medications may be inactivated (particularly acid-labile) (Lou et al., 2023). Second, drugs go through the first-pass metabolism, which may drastically reduce their bioavailability. Third, orally administered medications are exposed to changing conditions in the gastrointestinal tract (GIT) caused by the co-administration of different kinds of food or fluids. Intake of food or drink changes particularly pH values, which directly influence drug absorption, *e.g.* meal ingestion increases the gastric pH. In case of weakly basic drugs from BCS class II, increased gastric pH reduces the amount of a drug which can be absorbed (López Mármol et al., 2022). Depending on the properties of a drug, concomitant intake of a drug with food may lead to increased or decreased bioavailability (Koziolek et al., 2019).

Dosing conditions may affect drug behaviour and bioavailability, therefore, it is favourable to investigate them. The real-life dosing conditions in older adults and geriatric patients were investigated in our previous studies. The questionnaire study performed among German and Polish older adults revealed that the most commonly chosen fluids for medication intake were non‑carbonated water – tap water (30 % *vs.* 23 % of answers) and/or mineral (still) water (29 % *vs.* 28 % of answers), tea (14 % *vs.* 24 % of answers), carbonated water (10 % *vs.* 5 % of answers) and coffee (4 % *vs.* 5 % of answers) (*n* = 167 study subjects, *n* = 235 answers in total in Germany, *n* = 174 study subject, *n* = 292 answers in total in Poland) (Sarwinska et al., 2024, Sarwinska et al., 2025). Several studies have demonstrated that co-administered fluid (non−/low-caloric like green tea or caloric such as grapefruit juice or milk) influences drug and dosage form behaviour after oral administration (Jetter et al., 2002; Misaka et al., 2014; Chon et al., 2018). Mainly, the studies focused on pharmacokinetic interactions between the drug substance and a certain ingredient from the fluid, *e.g.* inhibition of enzymes or drug transporters, complex formation between an ingredient and a drug substance. There is limited literature data about the influence of intake conditions on a dosage form. For example, the *in vitro* study by Akshay et al. investigated the influence of 200 mL of different fluids (water, carbonated drink, milk and buttermilk, all at 20 °C, tea, and coffee, both at 50 °C) mixed with 700 mL of Simulated Gastric Fluid pH 1.2 on drug release (Akshay et al., 2021). The release of paracetamol from tablets was reduced in all media except water and phosphate buffer. The strongest effect was observed in tea. In addition to tablets, hard capsules are commonly used oral dosage forms. The study of Witticke et al. demonstrated that capsules were used by 21.8 % of study participants and were also commonly preferred dosage form (54.2 %) (Witticke et al., 2012). In another study, a total of 677 German participants were asked about their preferred dosage form for vitamin products. The results revealed that the capsules were chosen by 53 % of the participants (POSpulse, 2022). Capsules have several advantages over tablets *e.g.* they are a better formulation for substances with low compressibility, bitter taste or slow dissolution (Al-Tabakha, 2010). Capsules are one of the preferred dosage forms, which is also reflected in the economics. In 2023, the global market value of the pharmaceutical empty capsules for oral administration reached 1456.2 million U.S. dollars, representing approximately a 10 % increase since 2021 and it is foreseen to further increase in the future (Reports and Insights, 2023). Recent trend directs the production of capsules to plant-based products rather than animal-derived, due to health, religious or dietary concerns. That is also the case in capsule manufacturing, where instead of gelatine capsules other plant-based capsules are being developed *e.g.* hypromellose (HPMC) or pullulan (Industry Intel Connect Insights, 2024; Gren, 2023). The most common hard capsule shells are made of gelatine (hard gelatine capsules, HGC) or hydroxypropyl methylcellulose (HPMC, hypromellose). Both capsule types have different properties. It is generally known that gelatine capsules are sensitive to high temperatures (Majee et al., 2017). Moreover, the process of cross-linking reduces the solubility of gelatine, which directly influences the dissolution. This process may be induced by aldehydes or in stressed conditions such as high temperature and humidity (Lu and Shah, 2017). Conversely, gelatine capsules provide better protection against the permeability of oxygen than HPMC capsules (Junginger, 2020). In contrast, HPMC-only capsules, are plant-derived formulations based on the cellulose polymer. HPMC capsules are soluble in cold water but practically insoluble in hot water (Raymond et al., 2009). Additionally, the cross-linking was not reported for HPMC capsules (Majee et al., 2017). Moreover, HPMC capsules demonstrated more resilience to environmental conditions such as temperature and humidity, thereby providing better protection of the active pharmaceutical ingredient (API) (Yang et al., 2020). Several studies in the literature investigated the behaviour of capsules in different models and study designs (USP 2 apparatus, GastroDuo model, dynamic open flow-through test apparatus, *etc.*), mostly in water at RT or typical dissolution media (SGF, FaSSIF, *etc.*) (Sager et al., 2019a, Sager et al., 2019b; Schick et al., 2019; Garbacz et al., 2014). Furthermore, in PhysioCell, the temperature gradient of the dissolution medium was applied (Garbacz et al., 2014). However, the influence of real-life fluids such as tea or coffee on capsule shells has not yet been investigated *in vitro* or *in vivo*.

The presented study aimed to assess the influence of different co-administered fluids on capsules behaviour and disintegration *in vitro* as well as *in vivo*. The choice of fluids for the study is based on the results from the questionnaire study about drug intake in older adults and geriatric patients mentioned before in the introduction and includes tap water, mineral water, sparkling water, mint tea, black tea and coffee (Sarwinska et al., 2024, Sarwinska et al., 2025). First of all, we investigated the influence of different media on the capsule performance *in vitro* in a standard experimental set-up using USP 2 apparatus and the biorelevant model GastroDuo. Afterwards, we investigated the influence of different water temperatures (warm 50 °C and cold 8 °C) and warm black tea 50 °C on the capsule performance *in vivo* and on the gastric emptying utilizing the salivary tracer technique, where caffeine kinetics was used as a marker. In the end, we compared the *in vitro* and *in vivo* results to evaluate the performance of the capsules and to assess the predictive power and accuracy of *in vitro* tools.

## 2 Materials and methods

### 2.1 Capsules for *in vitro* experiments and clinical study

In this study, hard gelatine (Capsugel® Coni-Snap®, Capsugel Belgium, Bornem) and HPMC (Capsugel® Vcaps® Plus, Capsugel France, Colmar) capsules were used. Both capsules were size 0 and a natural transparent colour. A batch of powder mixture was prepared from the substances listed in Table 1. Each capsule (for *in vitro* and *in vivo* studies) was filled manually with 250 mg of the powder mixture with 1 % allowed variation. For the *in vivo* study, capsules contained the same ingredients, except caffeine, which was an isotope-labelled ^13^C_1_ caffeine.

**Table 1:** Composition of the powder mixture for capsule filling.

Ingredient	Manufacturer *In vitro/in vivo*		Quantity per one capsule (mg)	Quantity %
Caffeine (^12^C/^13^C_1_)	Caelo, Hilden, Germany	Eurisotop a Cambridge Isotope Laboratories Company, Cambridge, MA, USA	25.000	10.000
Microcrystalline Cellulose (Pharmacel 102)	DFE Pharma, Goch, Germany		223.875	89.550
Colloidal silicon dioxide, Aerosil® 200)	Fagron, Glinde, Germany		1.125	0.450

### 2.2 Coated tablets for evaluation of gastric emptying

The coated tablets consisted of a core and a pressed coating. The tablets were in size of 9 mm. The core contained 24 mg of ^13^C_3_ caffeine (Eurisotop a Cambridge Isotope Laboratory Company, Cambridge, MA). The tablets were manufactured by direct compression (Nagema KP2 eccentric tablet press (VEB Kombinat Nagema, Dresden, Germany)) from the powder mixtures prepared according to the protocol and utilizing the same substances from the study of Tzakri et al. (Tzakri et al., 2023)**.** Quality control of cores and pressed-coated tablets were performed and acceptance levels were reached (mean ± SD) (cores: breaking force (14 ± 4 N, *n* = 5), disintegration time (within 15 s, *n* = 3), weight (45 ± 2 mg, *n* = 20), height (2 mm, *n* = 10) and pressed-coated tablets: breaking force (87 ± 3 N, *n* = 4), disintegration time (within 20 s, n = 3), weight (265 ± 6 mg, n = 20), height (5 mm, n = 10), dissolution (300 mL SGF_sp_, 25 rpm, 37 °C) (> 90 % released within 5 min, n = 3)). Additionally, the pressed-coated tablets were tested in warm black tea (25 mL SGF_sp_, 240 mL black tea at 50 °C, 75 rpm, n = 3) to confirm the fast disintegration and caffeine release. The acceptance level was met. Pressed-coated tablets were also tested in warm water and cold water to confirm the homogenous behaviour and caffeine release (25 mL SGF_sp_, 240 mL water at 50 °C/8 °C, 75 rpm), n = 3 for every fluid). Fast disintegration and caffeine release were achieved in all fluids. Every tablet was packed separately in an aluminium bag (aluminium bags Ströbel GmbH (Langenzenn, Germany)). The details about the tablets' preparation and quality control can be found in the publication Tzakri et al. (Tzakri et al., 2023).

### 2.3 *In vitro* experiments

For the study, two *in vitro* tools were used: the pharmacopeial USP 2 apparatus and the GastroDuo biorelevant model.

#### 2.3.1 USP 2 apparatus

The dissolution experiments were performed in the pharmacopeial USP 2 apparatus (PT-DT8 USP II, Pharma Test Apparatebau AG, Hainburg, Germany). As a medium for the experiment, 25 mL of Simulated Gastric Fluid without pepsin (SGF_sp_) pH 1.2 (reflecting the gastric residual volume) and 240 mL of the tested fluid were used. The temperature of the dissolution apparatus together with vessels filled with SGF was set at 37 °C. The medium to be analyzed was set to its specific temperature: cold (8 °C), RT (20 °C) or warm (50 °C). The stirring rate was 75 rpm. Capsules for the study were placed in the spiral sinkers (12 mm). Samples were collected at defined time points. In USP 2 apparatus for gelatine capsules *n* = 3 for all tested fluids. For HPMC, *n* = 3 for cold water, water RT, mineral water, black tea, coffee, mint tea, and *n* = 6 for warm water, and sparkling water. The results from the last two fluids were variable, therefore, the experiment was repeated on a further 3 capsules.

#### 2.3.2 Gastro duo

**Fig. 1:**
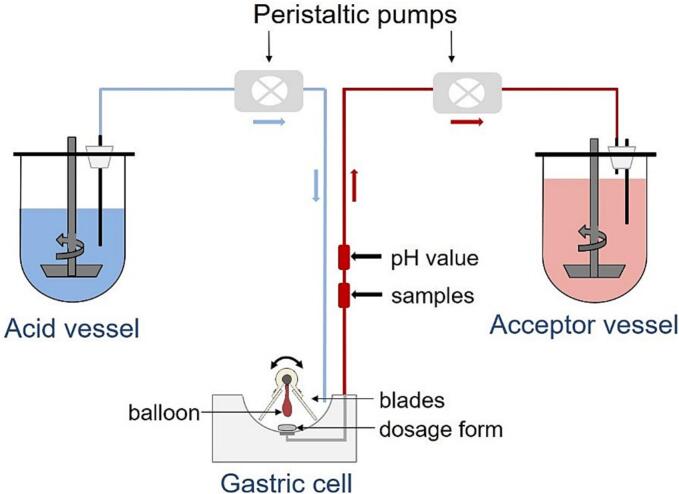
Schematic representation of GastroDuo, modified from Schick et al., 2019.

The second *in vitro* tool used in the study was the biorelevant GastroDuo model. The GastroDuo was used in order to simulate the disintegration, the dissolution and the gastric emptying in more biorelevant conditions than the USP 2 apparatus. This model is designed as a flow-through model cell and can simulate different physiologically relevant parameters influencing the gastric emptying process, like temperature, motility, dynamic pH changes and emptying rates (Schick et al., 2020). The schematic representation of the GastroDuo model is shown in Fig. 1. In GastroDuo, for gelatine capsules n = 3 for all tested fluids, except warm water, where *n* = 5. For HPMC, n = 3 for all fluids, except cold water, where *n* = 4. In GastroDuo, different number of samples were tested, due to variability in results and problems with pumps.

The dosage form is placed in the central compartment (gastric cell) between two blades and an inflatable balloon. The blades provide movement patterns, and the inflatable balloon simulates the pressure events. In the gastric cell, initially 25 mL of deionized water was used instead of SGF_sp_. This allowed simulating the appropriate pH values (5–7) *in vitro* that in real life would be obtained in the stomach after ingestion of 240 mL of water (Koziolek et al., 2015). Using only SGF_*sp*_ would not allow obtaining the desired real-life pH profiles*.* By using peristaltic pumps, the inflow of SGF_sp_ pH 1.2 can be controlled in order to simulate gastric secretion, which further ensures the proper pH profile.

By adjusting the outflow with different peristaltic pumps, the first-order-like gastric emptying of noncaloric fluid can be simulated. The sample port (1 mL) is installed at the outflow of the gastric cell to measure drug concentrations. Additionally, another sample port is installed in the acceptor vessel to measure the emptied amount of drug. Moreover, the model contains pH-electrodes (InLab®Expert Pro, METTLER TOLEDO, Greifensee, Switzerland) placed at the outflow and in the acceptor vessel to control the pH measurement and to characterize the media. For more details, we refer to a previous publication (Schick et al., 2020). The experiments were conducted in fasted-state conditions. The used media are presented in Table 2.

**Table 2:** Volumes and media types used in the GastroDuo model.

Location of the fluid	Type of fluid	Volume of fluid
Acid Vessel	SGF_sp_ pH 1.2	1000 mL
Gastric cell	Deionized water	25 mL
Ingested volume	Fluid to be tested	240 mL
Acceptor vessel	SGF_sp_ pH 1.2	575 mL

**Fig. 2:**
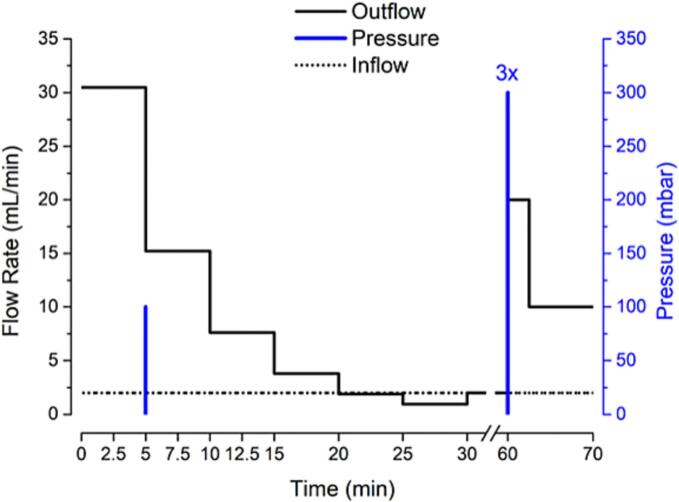
Schematic representation of the test program applied in the GastroDuo experiment.

At the start of the experiment, the gastric residual volume (25 mL) was adjusted to 37 °C, in order to simulate a realistic temperature profile. Then, 240 mL of the tested medium (at a certain temperature: warm, RT or cold) was transferred to the gastric cell with the gastric residual volume, resulting in 265 mL starting volume. After 5 min, a small pressure and movement event occurred to simulate small motility in the human stomach. The program used during experiments is shown in Fig. 2. By setting the inflow rate of SGF_sp_ pH 1.2 to 2 mL/min, the volume in the gastric cell was acidified to simulate a gastric secretion and by this, a realistic pH profile. By adjusting the outflow of the gastric cell, the first-order-like gastric emptying of noncaloric fluid was simulated in the first 30 min. The initial flow rate was 30 mL/min, which gradually decreased. It was halved every 5 min. After 30 min, the value was fixed. After this time, the in- and outflow were equal, to keep a constant volume in the gastric cell. After 60 min, a high pressure and movement pattern occurred, combined with high flow rates, to simulate the Migrating Motor Complex (MMC) phase 3 and to flush out the gastric cell. At predetermined time points 1 mL sample was taken and prepared for analysis. The pH was recorded every 10 s.

The main parameter to assess the capsules behaviour *in vitro* was the opening time. In both *in vitro* systems, it was assumed that the capsule opened when the measured caffeine concentration exceeded 15 μg/mL as described previously (Sager et al., 2019a, Sager et al., 2019b).

### 2.4 Analysis of samples from *in vitro* studies

Caffeine concentrations in water media in the USP 2 apparatus were measured in-line every minute by using a UV–Vis spectrophotometer (UV–Vis-Cary® 50 UV–Vis spectrophotometer (Varian Inc., Mulgrave, Australia)) equipped with a fiber optic system at a wavelength 272 nm (caffeine) and 500 nm (baseline correction). Fiber optics were equipped with removable tips with a gap width of 2 mm. The calibration range was 0.006 mg/mL to 0.115 mg/mL. The spectrophotometric measurements were not possible in the case of black tea, mint tea and coffee, which consist of several chemical ingredients and are not UV transparent. Therefore, the HPLC method for these complex fluids was developed and validated following the FDA guidelines in relation to accuracy, precision, specificity, linearity and range (U.S. Department of Health and Human Services Food and Drug Administration, 2018). The samples from black tea, mint tea and coffee were analyzed by using HPLC with UV/Vis detection with a calibrated range between 0.008 mg/mL and 2 mg/mL (R^2^ > 0.998). A Shimadzu Nexera XR HPLC system (Shimadzu Corporation, Kyoto, Japan) consisting of a DGU-20A5R degassing unit, two LC20ADXR solvent delivery modules, a Sil-20ACXR autosampler, a CTO-20 AC column oven and an SPD-M20A photodiode array detector was used. A Phenomenex Kinetex F5 column (150 × 2.1 mm, 2.6 μm), equipped with a precolumn (Phenomenex Inc., Torrance, CA, USA) was utilized. The mobile phase consisted of acetonitrile and water with 0.1 % formic acid (5:95, *v*/v). The time of analysis was 8 min, the retention time was 5.2 min and the injection volume was 1 μL. The temperature of the column was set at 40 °C. The elution was isocratic with a flow rate of 0.6 mL/min. The samples were measured by the UV–Vis detector on a wavelength 272 nm. For HPLC analysis, 1 mL samples from each vessel (USP 2) were collected. Every withdrawal sample was replaced with 1 mL blank medium. Before HPLC analysis, the samples of the complex media were diluted with SGF_sp_ pH 1.2 (1:1) and then centrifuged at 13000 rpm for 10 min. All used solvents and chemicals had analytical standards.

### 2.5 Media and fluids

In the study as a fasted state medium SGF_*sp*_ pH 1.2 was used. Fluids tested in the studies are presented in Table 3. Both types of tea were prepared by brewing one tea bag per one vessel/cup with 240 mL of boiling tap water. The brewing process lasted 2 min in the case of black tea and 4 min for mint tea. After this time, tea bags were removed from the vessel. Coffee was prepared by dissolving 3.0 g of coffee powder in 240 mL of boiling tap water.

**Table 3:** Media tested during experiments.

Medium	Manufacturer
Tap water	Stadtwerke Greifswald, Greifswald, Deutschland
Mineral still water	Volvic, Danone, Frankfurt am Main, Deutschland
Sparkling water	Glashäger, Bad Doberan, Deutschland
Black tea	PG Tips Original, Unilever UK Limited House, Surrey, UK
Mint Tea	TESCO PEPPERMINT INFUSION, Tesco Stores Ltd., Welwyn Garden City, England
Coffee	Kenco smooth, instant coffee, Jacobs Douwe Egberts GB LTD, Maidenhead, England

Since black tea and coffee contain caffeine, the amount of caffeine was assessed before the experiment on ten occasions, and a mean value was calculated. Black tea contained approximately 57.6 mg of caffeine in 240 mL (0.24 mg/mL) and coffee 96.0 mg (0.40 mg/mL). The caffeine concentrations of the media were considered as a blank sample and were subtracted from the measured values during the experiment. The pH values of the fluids were pH 6.3 for black tea, pH 7.0 for mint tea and pH 5.5 for coffee. In the study, the temperatures are considered as follows: cold 8 °C, room temperature (RT) 20 °C, and warm 50 °C.

### 2.6 Clinical study procedure

A clinical study was performed to assess the influence of different fluids on the capsules' behaviour *in vivo* and to assess the influence of water at different temperatures, as well as black tea on gastric emptying. In order to achieve these aims, the salivary tracer technique (STT) was implemented. The salivary tracer technique is a validated, non-invasive method that allows assessing the gastric emptying of liquids based on the kinetics of caffeine (Sager et al., 2018). The validation and details about STT are presented in the publication by Sager et al. (Sager et al., 2018). In our study, we used two different isotope-labelled caffeine ^13^C_1_ in capsules and ^13^C_3_ in pressed-coated tablets. The ^13^C isotopes are stable isotopes and are used to label the methylene groups of caffeine. Different isotopes differ in molecular weight, therefore, they can be measured by Liquid Chromatography with a Mass Spectrometry detector (LC-MS).

**Fig. 3:**
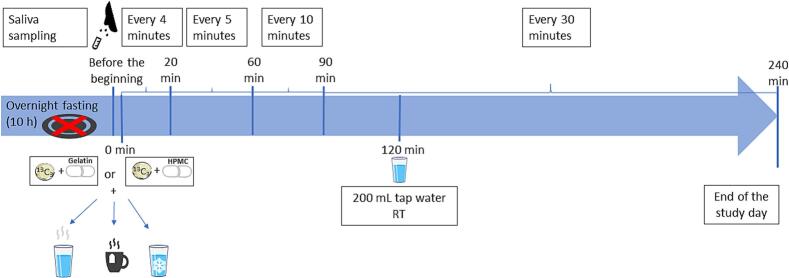
Schematic representation of the clinical study procedure.

The study had six arms (three fluids and two capsule types) and a cross-over design (Table 4). Every participant participated in all six study arms and was randomly assigned to a certain study day. Between study days, there was at least 72 h washout phase. After 10 h of overnight fasting, study participants received a capsule (HGC or HPMC) and pressed-coated tablet simultaneously with 240 mL of tested fluid (warm tap water (50 °C), cold tap water (8 °C) or warm black tea (50 °C)). Due to the complexity of the clinical study, not all fluids from the *in vitro* part were tested *in vivo*. Fluids for the clinical study were chosen after the *in vitro* study based on the strongest effects on capsule shells *in vitro*. The study procedure is summarised in Fig. 3. Due to the use of different types of isotope-labelled caffeine, it was possible to administer a capsule (with ^13^C_1_ caffeine) and a tablet (with ^13^C_3_ caffeine) simultaneously, also together with a black tea, which also contains natural caffeine (^12^C).

**Table 4:** Summary information about study arms.

Study arm	Administered dosage forms	Co-administered fluid 240 mL
A	gelatine capsule + pressed-coated tablet	Warm tap water 50 °C
B	HPMC capsule + pressed-coated tablet	Warm tap water 50 °C
C	gelatine capsule + pressed-coated tablet	Warm black tea 50 °C
D	HPMC capsule + pressed-coated tablet	Warm black tea 50 °C
E	gelatine capsule + pressed-coated tablet	Cold tap water 8 °C
F	HPMC capsule + pressed-coated tablet	Cold tap water 8 °C

### 2.7 Study participants

Study participants were recruited at the Department of Biopharmaceutics and Pharmaceutical Technology at the University of Greifswald. Gender balance was assured. Informed consent was obtained from all 12 healthy young volunteers involved in the study. The study was conducted following the Declaration of Helsinki, was approved by the ethical review board at the University Medicine of Greifswald, Germany (ethical protocol no. BB 161/23, 27.10.2023) and registered at the German Clinical Trials Register (Deutsches Register Klinischer Studien) (DRKS-ID: DRKS00033547).

### 2.8 Sampling, sample preparation and analysis

At certain time points, study participants were collecting saliva samples to 1.5 mL SafeSeal microtubes (Sarstedt, Nümbrecht, Germany) within 20 s. Prior to the intake of the dosage forms, a blank sample was collected. Collected samples were stored at −80 °C until analysis.

Prior to analysis, frozen samples were thawed at room temperature for about 1.5 h, then centrifuged for 15 min at 18000 *g* with a centrifuge SIGMA 3-30KS (Sigma Laborzentrifugen GmbH, Osterrode am Harz, Germany). 100 μL of the supernatant was transferred to 1.5 mL microtubes (Sarstedt, Nümbrecht, Germany) together with 200 μL of a solution containing acetonitrile and formic acid (94:6, *v*/v) to precipitate the proteins in saliva. Samples were frozen again. Calibration and quality control samples were prepared by using a blank saliva as a matrix. The blank saliva was free of caffeine of any isotope composition. All samples had the same matrix to compensate for the matrix effect and were also prepared identically. We also included both blank and double blank (without internal standard) samples in each measurement as a control. Additionally, the matrix effect was determined beforehand (also against pure solvent). All calibration, quality control and measurement samples (200 μL) were centrifuged at 3200 *g* for 5 min at room temperature. 150 μL of the supernatant was transferred to a new Eppendorf reaction tube and 300 μL of AcN / H_2_O (50:50, *v*/v) was added. Then, 10 μL of the internal standard 8-chlorotheophylline was added. 5 μL of samples was then injected to the chromatographic system.

Sample analysis was performed using LC-MS/MS, which includes Shimadzu Nexera LC-30 (Shimadzu Corporation, Kyoto, Japan) and the mass spectrophotometer QTRAP4000 (AB Sciex, Darmstadt, Deutschland). Calibrated range was between 5 ng/mL and 600 ng/mL (R^2^ > 0.999), and the LLOQ was the lower calibration value (5 ng/mL). The separation process was performed on the column Brownlee SPP RP-Amide (2.7 μm; 4.6 × 100 mm) with a SecurityGuard™ (C18; 4 × 2 mm) precolumn. Chromatographic separation was carried out under isocratic conditions, utilizing a mobile phase consisting of 0.1 % formic acid in ultrapure water and acetonitrile (70:30, v/v) at a flow rate of 0.6 mL/min. The retention time of different caffeine isotopes was 2.40 min for the caffeine ^13^C_1_ and ^13^C_3_. Electrospray ionization (ESI) was used for MS detection in positive ion mode. Ions were detected using the multiple reaction monitoring mode. The mass transition for ^13^C_1_ was 196.2 - > 139.0 (collision energy 25 V) and for ^13^C_3_ 198.2 - > 140.0 (collision energy 25 V). All chromatograms were evaluated according to the internal standard method using the respective peak areas. The concentrations were calculated using the peak area ratios to the internal standard with 1/x (x = concentration) weighted linear regression with the LC-MS software Analyst 1.7 (AB Sciex, Darmstadt, Germany).

Moreover, due to the occurrence of ^13^C_1_ caffeine in caffeine from natural sources and only a small difference in masses of ^12^C caffeine and ^13^C_1_ caffeine, high ^12^C caffeine concentrations in saliva could lead to falsely high signals of ^13^C_1_ caffeine but not of ^13^C_3_ caffeine. Thus, the standard solutions of all used caffeine types were also measured separately in all mentioned mass transitions to detect the detector's possible cross-interference signal for ^13^C_1_ caffeine, which was assessed to be 6.6 % of the ^12^C caffeine concentration. The data for ^13^C_1_ caffeine obtained in the study arms with black tea were corrected with the correction factor of 6.6 % of the measured ^12^C caffeine concentration at each time point according to the equation:

realc(C113caffeine)=measuredc(C113caffeine)−0.066×measuredcC12caffeine

This correction was also performed for datasets with a high initial concentration of natural caffeine in blank samples. Correction method was validated over a broad range of concentrations of ^12^C caffeine and ^13^C_1_ caffeine for five levels of ^12^C caffeine (100 ng/mL, 1000 ng/mL, 5000 ng/mL, 10,000 ng/mL and 15,000 ng/mL) and three levels of ^13^C_1_ caffeine (25 ng/mL, 300 ng/mL and 500 ng/mL). The chromatograms and the determination of the correction factor are presented in the supplementary materials (Fig. S5).

### 2.9 Data evaluation and statistical analysis

Pharmacokinetic parameters obtained from saliva sampling were the labelled caffeine appearance time (t_app_), the area under the curve (AUC), the time point of maximum concentration (t_max_) and the maximum concentration (C_max_). For AUC calculation, the trapezoidal method was used. Furthermore, AUC ratios for certain segments were calculated to compare the capsules behaviour and gastric emptying in different study arms (Eq. 1).

(1) $$\mathrm{AUC}\;\mathrm{ratio}\;\left(\%\right)=\frac{\mathrm{AUC}\left(0\to t\right)}{\mathrm{AUC}\left(0\to 240\right)}\times 100$$

t: 30 or 60 min.

AUC ratio allows the elimination of the effect of individual variability in caffeine exposure (Tzakri et al., 2024). A higher ratio indicates a faster rate of caffeine absorption and thus, gastric emptying.

Due to the small sample size, normal distribution was tested by two tests: the Kolmogorov-Smirnov test and the Shapiro-Wilk normality test. The results were assessed to be normally distributed when both tests were positive. A paired ANOVA with Dunns *post hoc* test was carried out to compare the data from different treatments within each group of capsules (A *vs.* C *vs.* E, B *vs.* D *vs.* F) and between all summarised tablet administrations with the same type of fluid (AB *vs.* CD *vs.* EF). The difference was considered significant when the *p*-value was <0.05.

For data handling and graphical compilation, Microsoft Excel (Version 2019, Microsoft Corporation, USA), and GraphPad Prism 9 (Version 9.5.1 (733), GraphPad Software, Boston, USA) were used. Statistical tests were performed with GraphPad Prism 9.

It was assumed that the capsules opened when the salivary caffeine concentration reached at least 25 ng/mL (5× LLOQ) and was gradually increasing.

## 3 Results

### 3.1 *In vitro* studies

#### 3.1.1 USP 2 apparatus

**Fig. 4:**
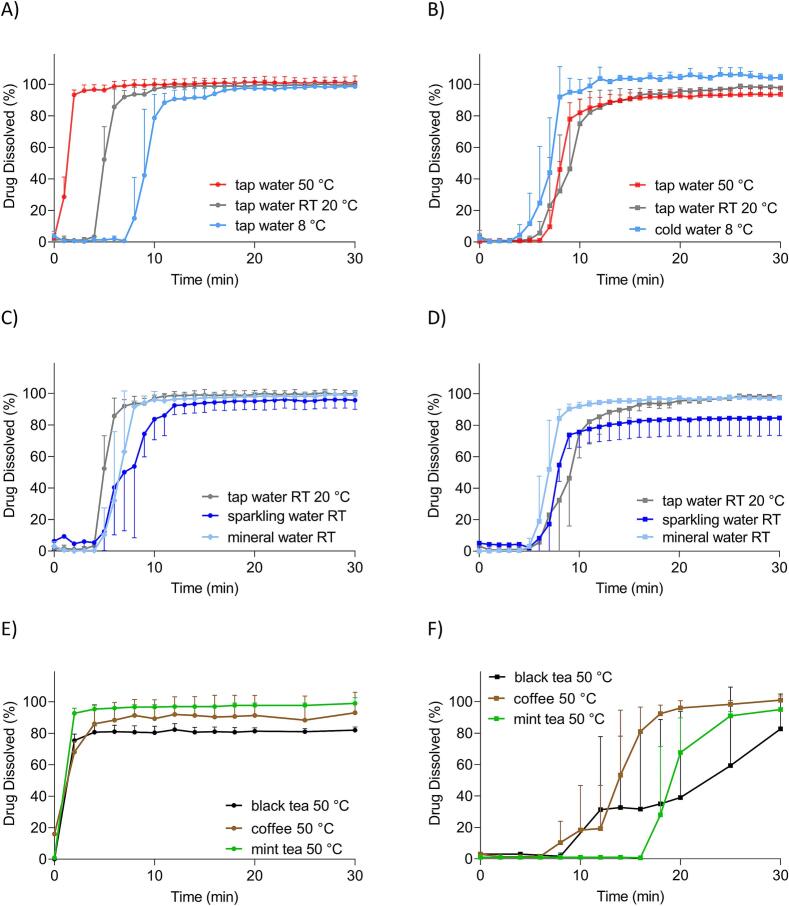
Results of the *in vitro* dissolution tests of gelatine capsules (left) and HPMC capsules (right) in water at different temperatures (A), (B), in different types of water (C), (D) and in media different from water (E), (F). All experiments were performed in the USP 2 apparatus. For gelatine capsules *n* = 3 for all experiments; mean +/− SD. For HPMC capsules n = 3 for all fluids, except mineral water RT, sparkling water RT, and tap water 50 °C, where *n* = 6; mean +/− SD.

In Fig. 4, the results from the USP 2 apparatus for both capsule types in different types of fluids are presented. The figure was divided into 3 parts, which focused on a different aspect: A, B – temperature effect; C, D – different water types; E, F – fluids different from water at 50 °C.

As can be seen, all gelatine capsules released more than 80 % of the caffeine in the first 10 min in all test set-ups. However, in black tea, within the whole experimental time maximum 80 % of caffeine was released. As shown, in the hot media, capsules opened immediately, and caffeine was released in the first 2 min (A and E). Moreover, the release was delayed with decreasing temperature (A). Different water types or media different from water did not change the release when compared to media with identical temperature profiles (C and E). It is worth noting that carbon dioxide could influence the measurement of the sparkling water in the UV–Vis, as higher standard deviations were observed in these experiments.

The results for HPMC capsules showed that all profiles had a lag time of at least 5 min. The opening time of the capsules in the USP 2 apparatus in cold water was a little bit shorter than in warm and RT medium, which were comparable (B). Although the release profiles in sparkling water did not reach 100 % (in contrast to mineral and tap water), results showed no difference in opening times of capsules in different water types (D). It should be noted that the variability of the total mass released is higher than the variability of the gelatine dissolution tests. As can be seen, the dissolution results of HPMC capsules in different media showed that the lag time and opening time increased in all media: coffee, black tea and mint tea, however, the most in mint tea (F). The release in coffee and mint tea reached almost 100 %, in black tea, however, approximately 80 %. After the experiment with HPMC capsules in black tea, the rest of the capsule shell was still present on the bottom of the vessel. The residues were in the form of a sticky brown mass on the sinker (Fig. 8).

The experiment in the USP 2 apparatus with HPMC capsules in black tea was repeated, however, with another medium, where SGF_sp_ was substituted with Fasted State Simulating Gastric Fluid (FaSSGF) and over a longer time (60 min). The results of this trial are available in supplementary materials (Fig. S1). It can be seen that the release was less affected, and full dissolution was achieved in approximately 30 min. Furthermore, the brown sticky residues were almost absent.

**Fig. 5:**
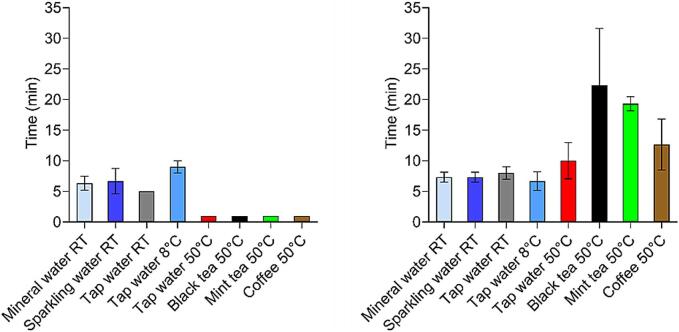
*In vitro* opening times of gelatine capsules (left) and HPMC capsules (right) in different fluids in USP 2 apparatus, *n* = 3 for gelatine capsules in every fluid, n = 3 for HPMC capsules in all fluids except mineral water RT, sparkling water RT and tap water 50 °C, where *n* = 6 for HPMC capsules. On the graph, the mean value ± SD is presented. For some fluids, no SD was presented, due to the same measured values. The opening time of the capsule was defined as the time point when the concentration of measured caffeine exceeded 15 μg/mL.

In Fig. 5, an overview of the opening times of both capsule types in the USP 2 apparatus in all tested fluids is presented.

#### 3.1.2 GastroDuo

**Fig. 6:**
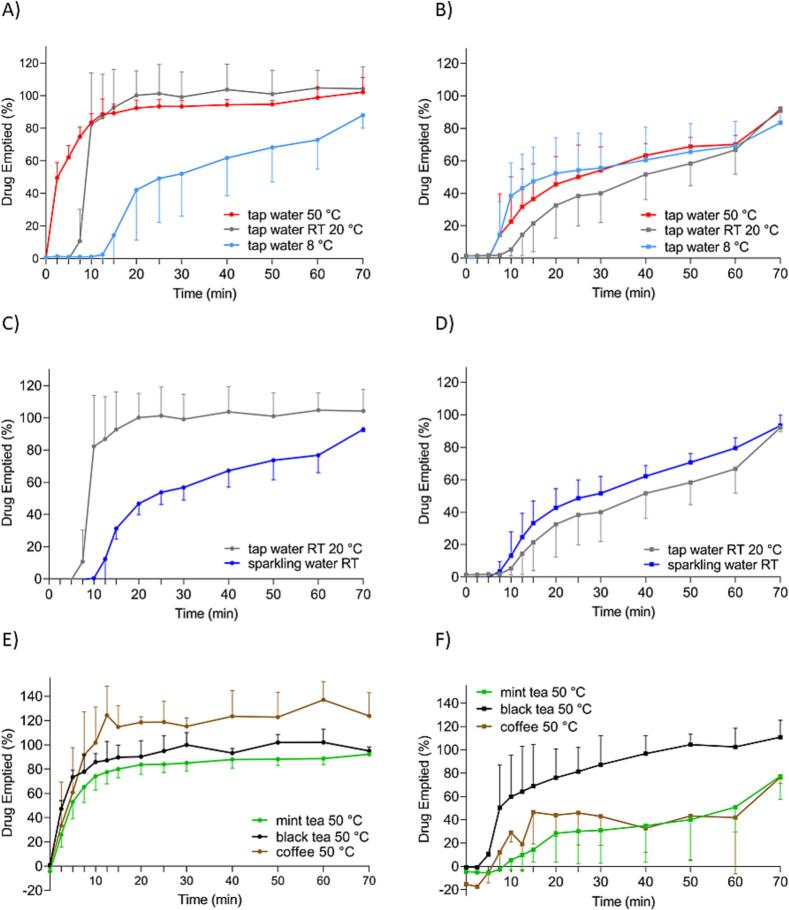
Amount of drug emptied to the acceptor vessel over the time of the dissolution tests of gelatine capsules (left) and HPMC capsules (right) in water at different temperatures (A), (B), in different types of water (C), (D), and in media different from water (E), (F). All experiments were performed in the GastroDuo system. For gelatine capsules, n = 3 for all tested fluids except tap water 50 °C, where *n* = 5, mean +/− SD. For HPMC capsules, n = 3 for all fluids except for tapwater 8 °C, where *n* = 4, mean +/− SD.

In Fig. 6, the results of the GastroDuo experiments, presented as the emptied amount of drug in the acceptor vessel, are shown. Similarly to the results from the USP 2 apparatus, the figure was divided into three parts, considering a certain aspect: A, B – temperature effect; C, D – different water types, E, F – fluids different from water at 50 °C.

As can be seen, hard gelatine capsules showed a fast emptying after administration with hot tap water compared to the other media (A, E). After simulating the small motility event after 5 min, the profile of the water at RT increased faster than the profile of cold tap water. After that, less than 80 % of the API was emptied after 60 min, in contrast, after administration with hot tap water and tap water at RT, more than 80 % of the API was emptied within 10 min. Compared to the administration with tap water at RT, the administration with sparkling water at RT showed a slower emptying process, more precisely, only 80 % of the API was emptied after 60 min (B). After administration of the gelatine capsule with other hot media (E), the amount of drug emptied increased very fast for all three media. After 15 min, more than 80 % of the API was emptied in each administration experiment. The amount of drug emptied after administration of the gelatine capsule with hot coffee reached levels of 120 % drug emptied. This may be related to the initial caffeine concentration of the coffee itself, which varied regardless of the consistent manner of preparing a brew. Conversely, in black as well as mint tea the amount of emptied drug was approximately 80 %.

The results of the HPMC dissolution test revealed that there was a lag time for each experimental setup. After 5 min, the concentration increased, the dissolution at every tested temperature was delayed and after 60 min, more than 60 % of the API had been emptied from the gastric cell (B). The administration of sparkling water resulted in a comparable dissolution profile to the profile after the administration of tap water at RT (D). In both cases, more than 60 % of the drug was emptied after 60 min. Media other than water at 50 °C demonstrated different release profiles (F). Only the dissolution profile after black tea administration resulted in more than 80 % of the emptied amount of the drug after 60 min. In the case of mint tea and coffee, it was less than 60 %. Interestingly, for black tea, the lag time was shorter. The lag time of both profiles was comparable to the other HPMC experimental lag times. Initially, in coffee the value of emptied drug - 20 % was reported, which comes from varying caffeine concentration in coffee.

**Fig. 7:**
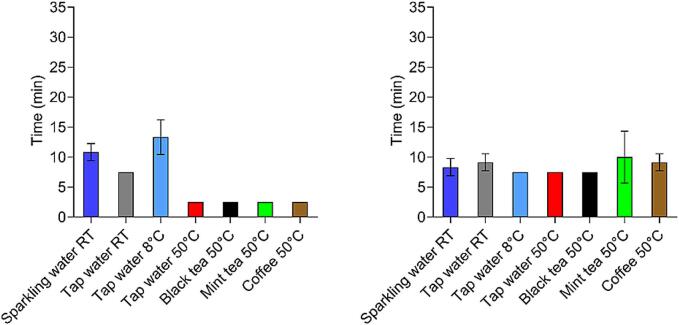
In vitro opening times of gelatine capsules (left) and HPMC capsules (right) in different fluids in GastroDuo, for gelatine capsules n = 3 for all tested fluids except for tap water 50 °C where n = 5, for HPMC capsules n = 3 for all fluids except for tap water 8 °C, where n = 4, on the graph, the mean value ± SD is presented. For some fluids, no SD was presented, due to the same measured values. The opening time of the capsule was defined as the time point when the concentration of measured caffeine in the outflow of the gastric cell exceeded 15 μg/mL.

In Fig. 7, an overview of the opening times in every tested fluid in GastroDuo is presented.

**Fig. 8:**
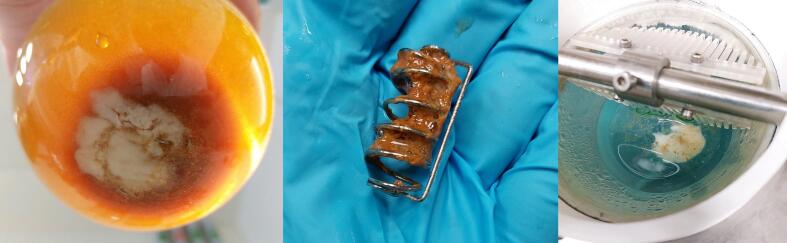
Residues after dissolution study in the USP 2 apparatus of HPMC capsules in the black tea and SGF in the vessel (left) and in the sinker (middle) and GastroDuo (right).

After the experiment in GastroDuo with HPMC capsules in black tea, capsule residues were also present in the gastric cell as in the USP 2 experiment. Most of the fluid was emptied from the vessel, and there was no sinker, however, the sticky residues of the capsule's shell were still present (Fig. 8).

**Fig. 9:**
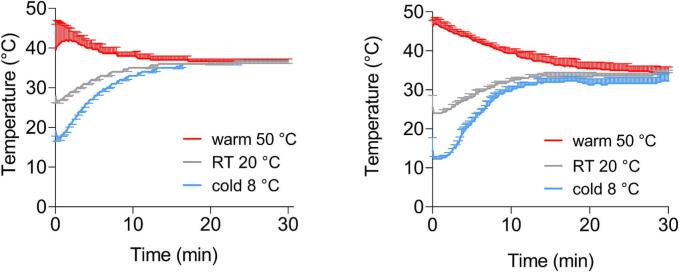
Temperature profile over time in the USP 2 apparatus (mean of n = 3 +/− SD) and GastroDuo (n = 3, mean +/− SD).

In Fig. 9, the temperature profiles of the experiments in the USP 2 apparatus and the GastroDuo are shown. In both systems, after 10 min, the warm media reached 37 °C, cold media and room temperature media after 18 min.

### 3.2 Clinical study with young healthy volunteers

Participants were recruited based on the inclusion criteria of age 18 to 64 years, Body Mass Index (BMI) between 18.0 and 29.99 kg/m^2^ and general good health. The intake of medications that affect the GIT (*e.g.* laxatives, antidiarrheal, opioids, antibiotics) was an exclusion criterion. In general, inclusion and exclusion criteria were in line with the EMA and FDA guidelines (EMA, 2010; FDA, 2002).

Overall, 12 young, healthy volunteers were recruited (6 women and 6 men). The summary of the study participant characteristics is presented in Table 5. All subjects successfully completed the study without adverse events or dropouts. All obtained saliva samples could be measured.

**Table 5:** Demographic data - summary.

Description	All study participants	Females	Males
Number of participants	12	6	6
Age in years (range, mean ± SD)	23–41, 26 ± 5	23–28, 25 ± 2	23–41, 27 ± 7
BMI in kg/m^2^ (range, mean ± SD)	19–30, 24 ± 3	19–28, 23 ± 3	22–30, 26 ± 3

For the data analysis of tablets, all tablet administrations were summarised (72 tablet administrations), thus, there were 24 administrations per fluid. During data analysis, an inexplicably high caffeine peak in an early phase was detected in 3 cases. In one case (C002), data could still be analyzed after the exclusion of the interfering peak. This contamination could have occurred during the sample preparation. In the rest two sample sets, the contamination could have occurred during tablet administration. For the final evaluation of the tablet data, the results from two participants were excluded due to contamination in the samples that disabled reliable data analysis. Finally, for the data analysis, 22 tablet administrations per fluid were taken into consideration (participant 007 was excluded from study arms with gelatine capsules A, C, E, and participant 002 was excluded from study arms with HPMC capsules B, D, F).

In order to determine whether individual t_app_ for a specific study arm (for capsules and tablets) were within the norm, the criterion of the sum of the mean value and twice the standard deviation was assumed. In 4 out of 72 capsule administrations and 1 out of 72 tablet administrations, unexpectable high t_app_ was measured that exceeded the given criterion. The individual t_app_ values of capsules for all study participants are available in supplementary materials (Table S4). Since the aim of the study was to investigate capsules properties *in vivo*, not pathological cases, therefore, in order to reduce the variability, the data for capsules were analyzed without the four mentioned cases. The study participant 004 was excluded from all study arms with gelatine capsules (A, C, E). In case of the study arms with HPMC capsules, participants 001 and 002 were excluded. Finally, *n* = 11 was achieved for gelatine capsules and *n* = 10 for HPMC capsules. Similarly, the data for pressed-coated tablets were analyzed after the exclusion of the extreme case (tablet administration of participant 001 from the study arms B, D, F). Finally, the *n* = 21 administrations per fluid are presented in the results section. Details about the excluded cases are presented in the discussion.

#### 3.2.1 Influence of different fluids on gelatine and HPMC capsules

**Fig. 10:**
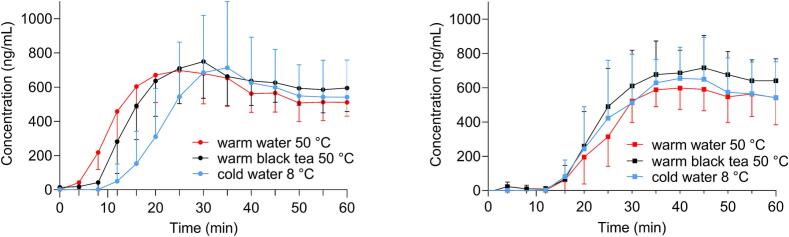
Mean profiles of the salivary caffeine concentrations for the first 60 min of gelatine capsules (left) and HPMC capsules (right) obtained after intake with the tested fluids at different temperatures, *n* = 11 for gelatine capsules in every fluid, *n* = 10 for HPMC capsules in each fluid (mean +/− SD).

Fig. 10 illustrates the mean profiles of salivary caffeine concentrations for both capsules in the tested fluids over the first 60 min. The salivary caffeine concentrations for both capsules in all fluids are presented for individual participants in supplementary materials (Fig. S4). Additionally, the summary graphs showing opening and appearance times, as well as release profiles from different methods used in the study, are presented in the supplementary materials (Figs. S2 and S3). In case of gelatine capsules, in the first 30 min, differences in the profiles in each fluid could be observed. The profiles in both warm media: water and black tea were similar with a highly delayed profile from cold water. After 30 min, cold and warm water profiles reached the same levels. In the case of HPMC capsules, all the profiles had a lag time of approximately 15 min. Mean profiles for each fluid were almost the same, with slightly higher exposure in black tea.

**Fig. 11:**
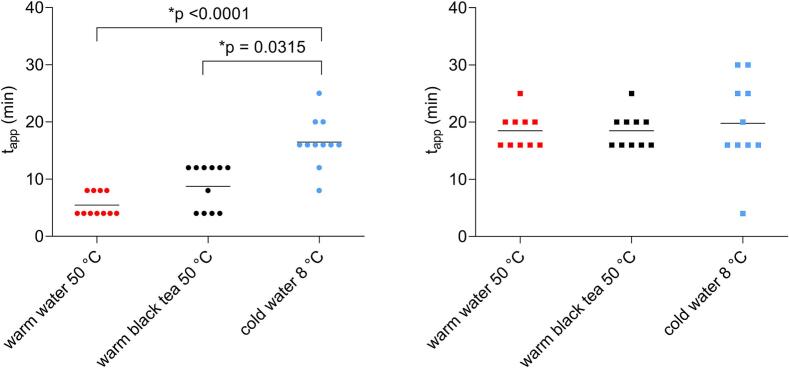
The scatter plot of t_app_ for gelatine capsules (left) and HPMC capsules (right) in tested fluids, n = 11 for gelatine in every fluid, n = 10 for HPMC capsules in every fluid. Individual data are represented as points and the mean value is depicted as a straight line.

The mean time required for gelatine capsules to open was found to be the longest in cold water (16.5 ± 4.4 min) and shortest in warm water (5.5 ± 2.0 min), with a significant difference between the two (*p* < 0.0001). The appearance time in warm black tea was slightly longer than in warm water (8.7 ± 3.9 min). In comparison to the t_app_ in cold water, a significant difference was also detected between warm black tea and cold water (*p* = 0.0315). For HPMC capsules, t_app_ in all tested fluids were similar and were approximately 20 min. There were no significant differences (Fig. 11).

**Fig. 12:**
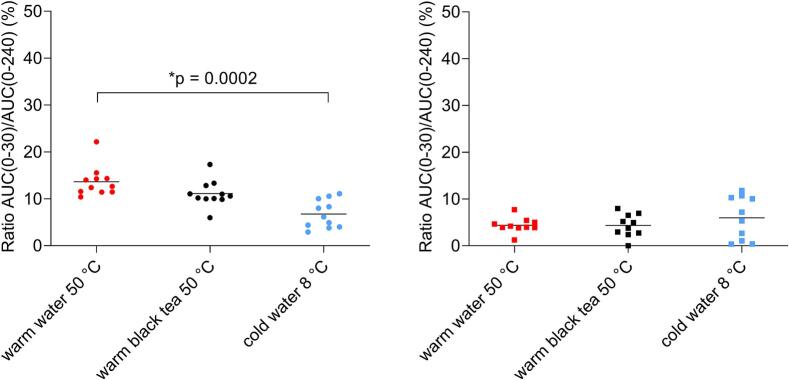
The scatter plot of AUC ratios AUC(0–30)/AUC(0–240) (%) for gelatine capsules (left) and HPMC capsules (right) in tested fluids, n = 11 for gelatine capsules in every fluid, n = 10 for HPMC capsules in every fluid. Individual data are represented as points and the mean value is depicted as a straight line.

**Fig. 13:**
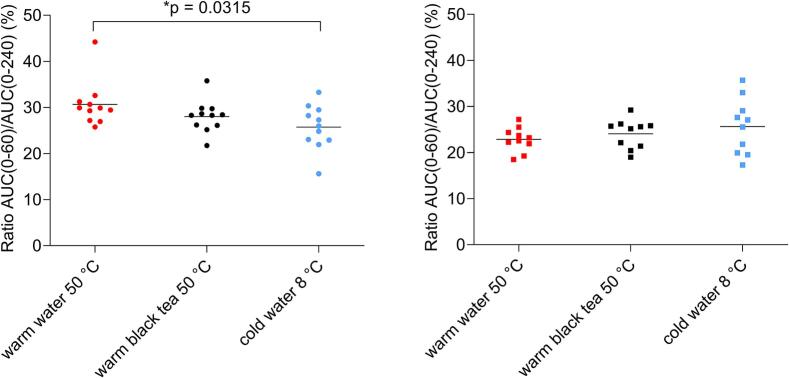
The scatter plot of AUC ratios AUC(0–60)/AUC(0–240) (%) for gelatine capsules (left) and HPMC capsules (right) administered with different fluids, n = 11 for gelatine capsules in every fluid, n = 10 for HPMC capsules in every fluid. Individual data are represented as points and the mean value is depicted as a straight line.

The highest t_max_ for gelatine capsules was achieved in cold water (40.9 ± 27.0 min) and the lowest in warm water (26.0 ± 6.0 min). For HPMC capsules, it was the opposite, the highest t_max_ was in warm water (51.0 ± 13.5 min) and the smallest in cold water (38.5 ± 11.6 min). In order to minimize the effect of intra- and interindividual variability in caffeine exposure, AUC ratios were calculated for certain segments: AUC(0−30)/AUC(0–240) and AUC(0–60)/AUC(0–240). The AUC ratios are presented in Fig. 12, Fig. 13.

In case of gelatine capsules, significant differences were observed in exposure between warm water and cold water for AUC ratios for both segments (*p* = 0.0002, p = 0.0315). For HPMC capsules, all calculated values were similar, and no significant differences were observed in both AUC ratios.

The tables with mean values of pharmacokinetic parameters (C_max_, t_max_, AUC) for both capsules are presented in the supplementary materials (Table S1, S2).

#### 3.2.2 Pressed-coated tablets - influence of different fluids on gastric emptying

**Fig. 14:**
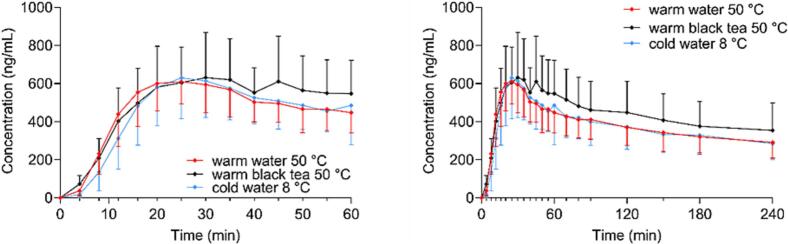
Mean profiles of the salivary ^13^C_3_-caffeine concentrations of tablets co-administered with different fluids, *n* = 21 administrations for every profile. For the first 60 min (left) and for 240 min (right), (mean +/− SD).

Fig. 14 depicts the caffeine kinetics from pressed-coated tablets administered with different fluids, which serves as a marker of gastric emptying. The salivary caffeine concentrations for all tablet administrations in all fluids are presented for individual participants in supplementary materials (Fig. S4).

The onsets of the profiles from warm water and warm black tea were almost the same, whereas the profile of the cold water was slightly delayed. However, after 25 min, all curves reached a point of convergence. Until the end, mean profiles for warm and cold water were identical. However, the profile for the administration of black tea revealed a slight elevation above the other two, indicating the highest exposure. There was a significant difference between t_max_ of warm water and warm black tea (*p* = 0.0208). The highest mean t_max_ was achieved in warm black tea (35.7 ± 12.0 min) and the lowest in warm water

(25.3 ± 9.4 min).

**Fig. 15:**
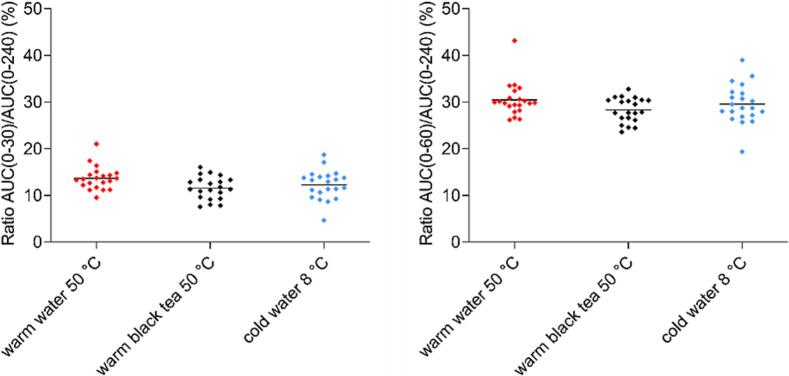
The scatter plot of AUC ratios (%) AUC(0–30)/AUC(0–240) (left) and AUC(0–60)/AUC(0–240) (right) in tested fluids, n = 21 tablets administrations for each fluid. Individual data are represented as points and the mean value is depicted as a straight line.

Similarly to capsule data, in order to hinder the inter- and intraindividual variability in caffeine exposure, AUC ratios for certain segments AUC(0–30)/AUC(0–240) and AUC(0–60)/AUC(0–240) were also calculated for tablets. Both calculated AUC ratios demonstrated no significant differences between administered fluids. The distribution of both AUC ratios is presented in Fig. 15.

The table with mean values of pharmacokinetic parameters (C_max_, t_max_, AUC) for tablets is presented in the supplementary materials (Table S3).

## 4 Discussion

The presented study aimed to investigate the influence of different fluids on gelatine and HPMC capsules *in vitro* and *in vivo,* as well as the gastric emptying of these fluids. The results demonstrated that the type of fluid influenced the behaviour and opening times of capsules *in vitro*, however, *in vivo,* the differences were mostly not significant. It is important to mention that assessing the opening time of capsules *in vivo* utilizing the salivary tracer technique is not a direct method. The parameter that is measured is appearance time (t_app_), which indicates when caffeine first appeared in the saliva samples. This time includes not only capsule disintegration but also several other events such as gastric emptying, absorption from the small intestine and distribution in saliva. All these events need time and therefore, they lead to a delay in the obtained value in comparison to the values obtained from the study, which utilized the direct method - Magnetic Resonance Imaging (MRI). According to Sager et al., the most important factors which influence the difference between measured concentrations in saliva and direct MRI measurements are gastric emptying and dissolution rate. The study also indicated that, on average, the time from capsule opening to caffeine appearance in saliva is about 4 min (Sager et al., 2019a, Sager et al., 2019b).

The results of *in vivo* measurements of capsules in the study arms with black tea were corrected with a correction factor to ensure accurate caffeine concentrations due to the occurrence of ^13^C_1_ caffeine in the natural caffeine and possible cross-interference of the detector. For the same reasons, the profiles from study participants with high initial natural caffeine concentrations in blank samples were also corrected this way. This allowed a reduction of the cross-interference and the obtaining of more reliable results. As mentioned in the results section, 4 out of 72 capsule administrations had abnormally high t_app_ values that met our exclusion criterion and therefore, were excluded. Because of the complexity of human physiology and the possibility of pathological events that may occur in the gastrointestinal tract, it is challenging to assess the reasons for the prolonged t_app_ of capsules and/or tablets. In three cases (D002, C004, B001), esophageal retention of the capsule or the so-called “lazy stomach” were suspected. After esophagal retention of the capsule, its disintegration, release and thus absorption even with regular gastric emptying are strongly delayed, whereas gastric emptying of dispersed tablet material is undisturbed, so that this caffeine profile shows fast absorption as expected. As known from recent MRI studies, the observation of esophageal retention is also not unlikely for HPMC based capsules (Rump et al., 2021). Interestingly, 2 out of 3 potential esophageal retentions of capsules happened while administered with black tea, where reduced drinking speed and higher temperature might provoke esophageal retention.

On the other site, the effect of a “lazy stomach” could also contribute to long t_app_. The “lazy stomach” is one of the gastric motility patterns reported by Romanski et al. and characterised by a late gastric emptying time, a slow gastric fluid flow and a gentle or delayed intragastric stress. The prevalence of the “lazy stomach” among participants from the mentioned study was 20 % (Romański et al., 2024). In our study, in the three mentioned cases, the curves for the administered pressed-coated tablets showed an immediate rise of the concentration as expected, which indicated typical gastric emptying of water, however, the t_app_ of the capsules was delayed. It demonstrated that the water with caffeine from the tablet was rapidly emptied into the duodenum; however, caffeine from the capsule had not yet reached the duodenum, or the capsule did not open although inside the stomach. Due to decreased mechanical stress of a “lazy stomach”, delayed disintegration could occur. In the case of HPMC capsules, stress events are prerequisite for disintegration. Therefore, we suspected that in cases B001, D002, the tiny stress events and small gastric fluid flow could have been strong enough to empty the caffeine from the fast-disintegrating tablet to the duodenum. However, it could not have been enough for the HPMC capsule to disintegrate and release its caffeine, so that it could not be emptied. Disintegration could have happened when the late housekeeping wave occurred, causing the release of the caffeine and emptying into the duodenum. In case of gelatine capsule, stress events are not essential for disintegration, therefore, the entrapment of the capsule in the esophagus could have had a stronger effect than a “lazy stomach”. Since it cannot be proven that atypical physiology led to the results which are aimed to present mainly capsule performance, complete data including these irregular data sets are given in supplementary files. As mentioned before, data from 2 volunteers from pressed-coated tablets were excluded due to sample contamination. In these cases, the contamination could have happened during tablet administration. Participants may have kept the tablet too long in the mouth, so it started to disintegrate there. In the profile, the carry-over was seen, which disabled the exclusion of any value and reliable data analysis. Therefore, the entire data sets for these participants were excluded. In one volunteer, abnormally high t_app_ was observed in both curves, for the capsule and the tablet (F001). This case reinforced the findings that there can be cases or individuals demonstrating a significant slowing of gastric emptying, even of non-caloric fluids. The reason for that could be the above-mentioned phenomenon of “lazy stomach” (Romański et al., 2024) or even something one might call “transient gastroparesis”. In the case of gastroparesis, dramatically reduced gastric emptying is observed (Camilleri et al., 2018). Typically, it occurs after food ingestion, however, the ingestion of even moderate amounts of water may also lead to a transient stop in gastric emptying. A similar case to one reported in our study was also observed in the literature (Paulick, 2013). In this study, non-caloric watery paracetamol (acetaminophen) solution was not emptied from the stomach within 30 min, showing a transient stop of water emptying in one healthy young subject out of 12 volunteers. Moreover, the plasma concentrations of paracetamol (BCS class I – good water soluble and good permeable) also showed no absorption during this time, so that a balance of emptying and secretion, which could also lead to an apparent stagnation in volume, could be excluded. This demonstrates that in some apparently healthy individuals, even the administration of moderate volumes of water may lead to gastroparesis or may be affected by this transient phenomenon, which seems to be even more pronounced than the aforementioned “lazy stomach”, although it could be an extreme case of it. Because the pressed-coated tablets are fast-disintegrating tablets, it is highly unlikely that they got entrapped in the esophagus, which could, in theory, also lead to slow absorption. After contact with water, they disintegrate within 20 s, immediately releasing the caffeine. Even on wet mucosal surfaces, the tablets disintegrate rapidly, so that they cannot be stuck in the esophagus for a relevant time, since it does not stay intact. In the study, participants ingested 240 mL of water, which would be enough to wet the tablet, begin the disintegration and therefore, the dispersion of caffeine, which would be further carried with water through the stomach to the duodenum. Thus, if both the profiles of caffeine from capsule and tablet show delayed absorption, this is probably rather related to slowed gastric emptying than esophageal retention of both dosage forms. The presented study aimed to investigate the capsule behaviour *in vivo* at different water temperatures and in black tea to learn more about their properties. We did not aim to observe physiological/pathological cases of capsule behaviour *in vivo* caused by several reasons. In order to investigate the influence of several phenomena such as esophageal retention, lazy stomach or gastroparesis, further studies should be conducted, including the imaging techniques *e.g.* Magnetic Resonance Imaging or gamma scintigraphy.

It is known that gelatine is thermosensitive and dissolves faster at higher temperatures (starting from 30 °C up) and that this phenomenon is less pronounced for HPMC, which dissolves independently of the temperature in the range from 10 to 55 °C (Raymond et al., 2009; Al-Tabakha et al., 2015; Chiwele et al., 2000). This was likewise observed in our results. In both *in vitro* systems, a strong influence of the temperature on gelatine capsules could be detected. In all warm media capsules opened immediately, because the temperature was above 30 °C. In case of coffee and black tea (USP 2) and black tea and mint tea (GastroDuo), complete release was not achieved, which could be due to possible analytical problems. Although the same tea and coffee were used for the preparation of the calibration curve and experiments, concentrations could fluctuate. *In vivo,* the strong temperature effect was also observed. Gelatine capsules opened the fastest in warm water, slightly slower in warm black tea, and took the longest time to open in cold water. It demonstrated that also *in vivo,* the temperature effect dominates over other influences.

Considering that the temperature was an important factor investigated in our study, it is worth noting how warm temperature for the study was chosen. Literature data suggests that the optimal temperature of warm beverages that was preferred or recommended by people was between 54 and 71 °C (Abraham and Diller, 2019). Other literature data suggested that drinking very hot beverages at a temperature above 65 °C was classified as a factor probably carcinogenic, causing especially esophageal tumors (Loomis et al., 2016). Additionally, prior to the study, a small-scale observation study was performed in order to assess the beverage temperature, which allows drinking a large amount (240 mL) of fluid at once. Participants were able to drink hot beverages at a temperature of 65–55 °C, however, mostly sip by sip. The temperature of 50 °C was chosen for our study, because that was the highest acceptable temperature of the fluid that could be drunk by people at once in a large amount (240 mL) without problems, discomfort or hazard. Moreover, the presented study focused on the temperature effect on capsules administered in real-life conditions, therefore, the temperature had to be adjusted to the real-life situation. Even though the higher temperature could be used *in vitro*, to obtain accurate results, the same temperature would have to be used *in vivo*.

The use of the fluid at higher temperature can be considered from several perspectives. Considering study participants, the higher temperature of the fluid could put patients at risk of burns and discomfort. Moreover, people who are more susceptible to higher temperatures would not be able to complete the study. The influence of the high temperature on the capsules' shells is crucial for gelatine-based capsules, since gelatine is thermosensitive. The higher temperature applied to gelatine capsules could accelerate the capsules' disintegration even more and cause the capsule disintegration already in the mouth. HPMC capsules in our study were not affected by the higher temperature, therefore, the same is expected also at even higher temperatures. The influence of higher temperatures on capsules shells can be further investigated, however, it will not be the real-life situation.

The study by Sun et al. demonstrated that the administered fluid loses its temperature rapidly. Interestingly, the intragastric temperature after the administration of 400 mL of fluid at 50 °C was reported to be approximately 44 °C directly after fluid administration (Sun et al., 1988). This demonstrates that the drink administered at a higher temperature will not ensure the same initial temperature when reaching the stomach, however, it would put patients at risk of burns and discomfort.

When ingesting 400 mL of a warm or cold drink, the intragastric temperature reaches 37 °C after approximately 20–30 min (Sun et al., 1988). In our study, participants ingested 240 mL of fluid, which is slightly more than half of the volume from the presented study. Furthermore, the cold temperature in our study was higher than in the work of Sun et al. Therefore, it can be assumed that in our study, physiological temperature was achieved faster than 30 min, especially in the case of a cold drink. Additionally, the study by Schneider et al. that used Smartpill® reported that after administration of 240 mL of RT water, the temperature rises from 22 °C up to 36 °C in approximately 20 min (Schneider et al., 2016).

In our study, mineral and tap water were considered as different water types, however, in some cases, the mineral water is just bottled tap water. In our study, we decided to use common mineral water and to investigate it against normal water from the tap and sparkling water, which is also mineral water but with carbonic acid. The differences between the water types are based on different amounts of minerals/ions (*e.g.* bicarbonates) and carbon dioxide (CO_2_). For gelatine capsules, differences in opening times and release profiles in all water types were not notable using the USP 2 apparatus. The release in sparkling water was delayed and slower than in tap water. During the experiment with gelatine capsules in sparkling water in GastroDuo, it was observed that the capsules were floating on the surface of the medium in the gastric cell. Due to this issue, the capsule had no constant contact with the fluid and therefore, the release could have been slower. As the rate of emptying from the gastric cell was gradually decreasing (1st order kinetics), the concentration in the acceptor vessel was gradually increasing. It is assumed that a similar situation may be present *in vivo* when the capsules float and stick to the stomach wall with reduced access to the fluid.

For HPMC capsules, the temperature effect was much more pronounced in the USP 2 apparatus compared to the GastroDuo model and the *in vivo* data. In GastroDuo, the observed opening times were almost identical for all tested fluids. *In vivo* data also showed comparable results for all fluids.

HPMC capsules behaved almost the same in tap water and in sparkling water in both *in vitro* systems. In the GastroDuo the release in sparkling water was slightly faster than in tap water. The carbon dioxide present in the medium may form bubbles on the surface of capsules and thus, alter the surface tension, which may influence the dissolution (Amaral Silva et al., 2019). Moreover, when HPMC capsules floated on the medium's surface, the presence of carbon dioxide might increase the mechanical stress, which is particularly important in the case of HPMC capsules rupture. Generally, in the GastroDuo the profiles were more gradual than in the USP 2 apparatus. In the USP 2 apparatus, the release in sparkling water was not complete, however, there was a high standard deviation, which could be because of the collection of air bubbles on the fiber optics.

Opening times of HPMC capsules in USP 2 apparatus in fluids different from water were higher, especially in black tea and mint tea. In the GastroDuo, release was delayed and reduced, especially in mint tea and coffee. Additionally, the negative values in the case of coffee were reported. Tea and coffee for experiments were always prepared following the standardized instructions, and the concentration was then measured on ten occasions. An average value was calculated and used as a baseline, which was then subtracted from each measured concentration value in the stomach cell. It should be noted that due to the naturally fluctuating caffeine content in black tea and coffee, and a flow-through type of system, differences in the caffeine concentrations of each sample are possible. The possible solution for this limitation could be a determination of the caffeine concentration in the individual brew and using it as a baseline for the whole process. The release profile in black tea in the GastroDuo was comparable to the results observed in a study by Akshay (Akshay et al., 2021). The literature data reported that about 262 mg of polyphenols (presented as gallic acid equivalent) are contained in a cup (230 mL) of a black tea infusion (Rechner et al., 2002). The reason for the delay and reduction in drug release from HPMC capsules could be the formation of complexes between HPMC and catechins (polyphenol compounds). A similar interaction between HPMC and catechins has already been reported and it also resulted in delayed release from HPMC capsules or decreased solubility of methylcellulose films *in vitro* (Glube et al., 2013; Yu et al., 2015). We assumed that *in vivo,* the interaction between HPMC and catechins would not be observed, due to the natural appearance of emulsifying substances in the GIT. The experiment with HPMC capsules in the USP 2 apparatus with black tea repeated with FaSSGF demonstrated faster and less variable release. FaSSGF has a pH value of 1.6, and contains taurocholate, phospholipids, which are surfactants and therefore they affect the surface tension. There are several ways in which they can optimise the drug behaviour, mostly by the influence on solubility, wetting, and diffusion coefficient (Bakatselou et al., 1991). The study by Vertzoni et al. demonstrated that the data from the solubility assessment of four poorly soluble medications were more accurate to predict the drug behaviour in the fasted stomach in FaSSGF, therefore, the use of that medium should be recommended for *in vitro* studies (Vertzoni et al., 2007). Since it was hypothesized that the interaction of catechins and HPMC formed the poorly soluble complexes, the use of biorelevant medium could dissolve the complexes and thus gave more accurate results. Tea has a complex matrix, therefore, the usage of biorelevant medium may be a more favourable choice for *in vitro* studies to obtain more accurate results.

*In vivo*, no significant impact of black tea on capsule opening times was observed. The average opening times for the three tested fluids were comparable and were approximately 20 min. For HPMC capsules, in the clinical study, no significant differences between any treatment were observed, which demonstrated that HPMC capsules are more robust and resistant to influences from tested fluids or temperatures *in vivo* than gelatine capsules.

Mint tea (*Mentha x piperita L.*) is also a mixture of several biologically active substances including essential oils, and polyphenols like flavonoids, which are responsible for its physiological effects (McKay and Blumberg, 2006). The literature reported amounts of 182.2 mg of polyphenols in one cup (250 mL) (Fecka and Turek, 2007). It can be assumed that, similarly to black tea, polyphenols from mint tea interfered with the dissolution of HPMC capsules *in vitro*. However, until now, there is no literature data about the influence of mint tea on the dissolution of capsules or other dosage forms *in vitro* or *in vivo*. However, it can be hypothesized that, also in this case, the effect would not be so pronounced *in vivo* as it was *in vitro.*

Similarly to both types of tea, coffee (*Coffea arabica L.)* is also a mixture of several compounds *e.g.* tannins, proteins, lipids, carbohydrates, acids, and polyphenols (such as chlorogenic acid) that can interfere with the drug release (Sharma, 2020). The literature data reported an average amount of 137 mg of polyphenols in 100 mL of a certain type of coffee brew (which corresponds to the content of approximately 328.8 mg in one cup of 240 mL) (Wierzejska et al., 2024). In our study, results demonstrated that coffee affected the *in vitro* release from HPMC capsules more than from gelatine capsules.

Since the temperature in the fasting stomach after the intake of water at RT reaches within a few minutes body temperature, the conditions are favourable for gelatine capsules (Koziolek et al., 2015). The administered warm media further promoted the release from gelatine capsules. Although the opening of HPMC capsules is independent of the temperature, the factor that plays a role here is mechanical stress. The influence of mechanical stress was observed, for example, in the study by Garbacz et al. where simulated pressure events promoted the opening of HPMC capsules (Garbacz et al., 2014). In our study in the USP 2 apparatus, there were no special pressure events. The only forces were generated by paddle movements. However, the program implemented in the GastroDuo model ensured a single pressure event after 5 min, which aided the opening of the capsules. Furthermore, there are also pressure events in the stomach, which are favourable for HPMC capsules. Moreover, HPMC has swelling properties and after placing it in the solution, it forms a hydrogel on the surface. The hydrogel layer delays the release of the API since the hydrogel layer dissolves gradually based on the diffusion mechanism (Douroumis, 2012; Kima Chemical Co., L, 2024). Therefore, the presence of pressure events is especially important to aid the rupture of the shell with the hydrogel layer. Considering all mentioned factors, the general appearance of release profiles from HPMC capsules is more gradual than in the case of gelatine capsules, where high temperature caused immediate release of caffeine and a steep slope of the profile.

The presented GastroDuo study aimed to investigate the influence of a motility event on the dosage form while it is still relatively intact. It is necessary to investigate the influence of a motility event without allowing prolonged contact with the warm liquid. It is unknown in which phase of the MMC the capsule is taken during administration *in vivo*. Therefore, the aim was to simulate an early stress event *in vitro* (after 5 min) to determine whether the capsules are generally susceptible to motility. In the subsequent phases of the investigation, the impact of elevated temperatures increases, which will, in turn, determine the susceptibility of the capsule to a stress event because the level of hydration rises. Therefore, it would be challenging to differentiate between the effects of temperature and motility on capsule opening. Conversely, applying a stronger stress event or later intermittent stress events could lead to rapid or inhomogeneous HPMC capsules disintegration and the release of the API. Additionally, it would not be possible to assess which factor, temperature, fluid ingredients or simulated motility caused the capsule rupture. The influence of different pressure events on the disintegration of capsules can be further investigated. The results from the GastroDuo demonstrated the same, homogenous behaviour of HPMC capsules as *in vivo,* which demonstrated that the implemented program was able to simulate a real-life situation.

In the study by Sager et al. behaviour of gelatine and HPMC capsules administered with 240 mL tap water at RT was investigated (Sager et al., 2019a, Sager et al., 2019b). The results demonstrated for tap water at RT the initial disintegration time (iDT = t_app_) of 15.5 ± 4.3 min for gelatine capsules, which is a similar value to our results in cold water (16.5 ± 4.4 min). There was a notable difference between t_app_ in RT water and warm water from our study (5.5 ± 2.0 min), which again demonstrates the pronounced influence of high temperature on gelatine capsules, also *in vivo*. The initial disintegration time for HPMC capsules reported by Sager et al. was 21.9 ± 12.1 min, which is a comparable value to our results in each medium, which also reinforced the robustness of HPMC capsules' behaviour in water at different temperatures.

The results from the presented study demonstrate that the fluid co-administered with capsules influence their opening times, and, therefore, drug release. In the case of gelatine capsules, the administration with cold water seems not to be much different from the administration with water at RT. Conversely, administration of gelatine capsules with warm water or warm black tea may accelerate drug release, leading to possible capsule opening even in the mouth. Administration of gelatine capsules with warm water was suggested by Chiwele et al. (Chiwele et al., 2000). Furthermore, HPMC capsules, since the temperature of administered water did not influence the opening times, can be administered with fluids in a wider range of temperatures.

It can be observed that in study arms with black tea, the total exposure to ^13^C_3_ caffeine from tablets was the highest in comparison to study arms with water. However, there was also a high variability in caffeine concentrations in all study arms. The literature data demonstrated high variability in caffeine pharmacokinetics in the general population, due to the polymorphism of cytochrome P450 enzymes, which is responsible for the metabolism of 95 % of administered caffeine (Nehlig, 2018). Therefore, AUC ratios of the main absorption phase (first 30 and 60 min) to the total exposure were calculated to adjust the effect of differences in caffeine exposure. The calculated AUC ratios did not show significant differences in the gastric emptying rates of water at different temperatures (50 °C and 8 °C) and of black tea (50 °C). The release profiles of pressed-coated tablets demonstrated a slight delay in the first minutes in case of cold water in comparison to warm media, however, there were no significant differences. This finding demonstrated that gastric emptying did not influence the behaviour of capsules and that differences in their behaviour are the result of their properties and the influence of co-administered fluids. Literature data demonstrated mostly a decrease in the gastric emptying rate or motility after administration of a cold drink. Sun et al. investigated the influence of different temperatures on the gastric emptying of fluids. The results yielded that cold (4 °C) and warm (50 °C) drinks (400 mL) emptied more slowly from the stomach than a drink at a control temperature (37 °C). Moreover, the initial gastric emptying rate in a cold drink was significantly slower in comparison to the control drink, which was correlated with differing gastric temperatures. The cold drink needed more time than warm drink to reach the physiological temperature (Sun et al., 1988). Fujihira et al. investigated the influence of the water temperature on gastric motility (Fujihira et al., 2020). The results demonstrated that the gastric contractions were hindered after the administration of 500 mL of cold (2 °C) water in comparison to control (37 °C) and warm (60 °C). Both studies, however, had a different design from our study, used a much larger volume of fluid, and a much lower temperature of the cold drink, which could cause a stronger response from the organism. Furthermore, in the study by Sun et al. a caloric liquid was used (orange juice), which will also influence the gastric emptying rate. Therefore, it can be assumed that the effects observed in the mentioned studies were much stronger than in our study, where no effect of the low temperature on gastric emptying was observed.

Considering the effect of black tea on gastric emptying or motility, the literature data are limited and often contradictory. The study by Chaudhuri and colleagues reported the prokinetic effect of black tea on the GIT in mice, where the gastrointestinal transit was significantly accelerated (Chaudhuri et al., 2000). Moreover, other resources demonstrated the antidiarrheal effect of black tea or the dose-dependent effect of decreasing or increasing gastrointestinal motility (Doustfatemeh et al., 2017; Besra et al., 2003; Jafari et al., 2006). More studies are needed to assess that influence on the GIT. Moreover, in contrast to water, black tea has a calorific value of approximately 2.5 kcal in 240 mL, however, such a small value seems to not influence gastric emptying (U.S. Department of Agriculture, 2019). Furthermore, the pH value of the fluid influences the gastric emptying. The study by Chaw et al. demonstrated a significant delay in the beginning of gastric emptying and a prolonged gastric residence time at pH 3 in comparison to the pH 7 buffer. In our study, the pH of administered black tea was approximately 6.3, which is slightly acidic and still close to neutral, therefore also the pH value of black tea seems to not influence gastric emptying.

## 5 Conclusion

Our results demonstrate that *in vitro*, real-life fluids had a more pronounced effect on capsule shells than *in vivo*. However, GastroDuo, as a biorelevant model, exhibited better predictive power than the USP 2 apparatus. *In vivo*, capsule intake with water at different temperatures and black tea had no considerable influence on the release of caffeine from HPMC capsules, whilst gelatine capsules were affected by the temperature in terms of the opening time. Additionally, no significant differences in gastric emptying of warm and cold water or black tea were observed. These findings demonstrated that gelatine capsules, also *in vivo*, are more susceptible to the temperature effects of co-administered fluid than HPMC capsules.

## CRediT authorship contribution statement

**Dorota Sarwinska:** Writing – review & editing, Writing – original draft, Visualization, Validation, Project administration, Investigation, Formal analysis, Data curation. **Mathilde Leyh:** Writing – review & editing, Validation, Investigation, Formal analysis, Data curation, Conceptualization. **Constantin Foja:** Writing – review & editing, Writing – original draft, Validation, Methodology. **Theodora Tzakri:** Writing – review & editing, Resources. **Philipp Schick:** Writing – review & editing, Supervision, Methodology, Conceptualization. **Felix Morof:** Writing – review & editing, Resources, Formal analysis. **Julius Krause:** Writing – review & editing, Resources, Methodology. **James Mann:** Writing – review & editing, Supervision, Resources, Conceptualization. **Richard Barker:** Writing – review & editing, Supervision, Resources, Conceptualization. **Mladen Vassilev Tzvetkov:** Writing – review & editing, Resources. **Werner Weitschies:** Writing – review & editing, Resources, Project administration, Funding acquisition, Conceptualization. **Michael Grimm:** Writing – review & editing, Supervision, Project administration, Methodology.

## Declaration of competing interest

The authors declare that they have no known competing financial interests or personal relationships that could have appeared to influence the work reported in this paper.

## Data Availability

Data will be made available on request.

## Appendix A Supplementary data

The supplementary materials contain all additional figures and tables referenced in the manuscript, supporting the results. The file is provided as a PDF. Image 1
