# Supplementary material for: The effect of black tea and water temperature on the disintegration of gelatine and HPMC capsules, tested with the paddle device, GastroDuo and in vivo pharmacokinetics: Much ado about little
Source: Int J Pharm X. 2025 May 27;9:100342. doi: 10.1016/j.ijpx.2025.100342 (PMC12166440; doi:10.1016/j.ijpx.2025.100342)
Supplement: Supplementary file 1 — The supplementary materials contain all additional figures and tables referenced in the manuscript, supporting the results. The file is provided as a PDF. [file mmc1.pdf]

Supplementary material to the publication:

The effect of black tea and water temperature on the disintegration of gelatine and HPMC capsules, tested with the paddle device, GastroDuo and in vivo pharmacokinetics: Much ado about little.

Dorota Sarwinska[1], Mathilde Leyh[1], Constantin Foja[1], Theodora Tzakri[1], Philipp Schick[1], Felix Morof[2], Julius Krause[1], James Mann[3], Richard Barker[4], Mladen Vassilev Tzvetkov[2], Werner Weitschies[1], Michael Grimm[1*]

1 Center of Drug Absorption and Transport, Department of Biopharmaceutics and Pharmaceutical Technology, Institute of Pharmacy, University of Greifswald, Felix-Hausdorff-Str. 3, 17489 Greifswald, Germany;

2 Department of Clinical Pharmacology, University Medicine Greifswald, 17487 Greifswald, Germany;

3 Oral Product Development, Pharmaceutical Technology & Development, Operations, AstraZeneca, Macclesfield, UK,

4 New Modalities & Parenteral Development, Pharmaceutical Technology & Development, Operations, AstraZeneca, Macclesfield, UK

*corresponding author

Table S1: *Pharmacokinetic parameters for gelatine capsules administered with different media in vivo, n = 11 for all fluids.*

	**Medium**		
**Parameter**	**warm water 50 °C**	**warm black tea 50 °C**	**cold water 8 °C**
**t_{max} (min)**	26.0 ± 6.0	31.8 ± 9.0	40.9 ± 27.0
**C_{max} (ng/mL)**	756 ± 175	893 ± 338	765 ± 370
**AUC(0-30) (ng × min/mL)**	14014 ± 3257	11614 ± 4220	6644 ± 4032
**AUC(0-60) (ng × min/mL)**	31401 ± 6099	30361 ± 6937	25242 ± 10517
**AUC(0-240) (ng × min/mL)**	103784 ± 20475	111671 ± 24895	98921 ± 34133

Table S2: *Pharmacokinetic parameters for HPMC capsules administered with different media in vivo, n = 10 for all fluids.*

	**Medium**		
**Parameter**	**warm water 50 °C**	**warm black tea 50 °C**	**cold water 8 °C**
**t_{max} (min)**	51.0 ± 13.5	45.5 ± 12.8	38.5 ± 11.6
**C_{max} (ng/mL)**	669 ± 103	825 ± 202	757 ± 179
**AUC(0-30) (ng × min/mL)**	3919 ± 2110	4890 ± 2942	4197 ± 3605
**AUC(0-60) (ng × min/mL)**	20904 ± 3890	23673 ± 7766	20822 ± 7096
**AUC(0-240) (ng × min/mL)**	93344 ± 18428	104458 ± 17462	90181 ± 24582

Table S3: *Pharmacokinetic parameters for pressed-coated tablets administered with different media in vivo, n = 21 for every fluid.*

	**Medium**		
**Parameter**	**warm water 50 °C**	**warm black tea 50 °C**	**cold water 8 °C**
**t_{max} (min)**	25.3 ± 9.4	35.7 ± 12.0	30.3 ± 13.6
**C_{max} (ng/mL)**	671 ± 163	735 ± 244	700 ± 200
**AUC(0-30) (ng × min/mL)**	12199 ± 2963	12197 ± 3206	10546 ± 2852
**AUC(0-60) (ng × min/mL)**	27296 ± 5709	29370 ± 8691	26586 ± 7347
**AUC(0-240) (ng × min/mL)**	90676 ± 20591	104515 ± 32603	90015 ± 23387

Table S4: *Opening times (min) for gelatine and hypromellose capsules from n = 12 participants and after the exclusion, in the fasted state in different media warm water, warm black tea and cold water. Data excluded from the analysis are marked with excl and/or certain colour, which symbolise the possible reason for the exclusion.*

**Volunteer**	**Gelatine capsules**			**Hypromellose Capsules**		
	**Warm water 50°C**	**Warm black tea 50°C**	**Cold water 8°C**	**Warm water 50°C**	**Warm black tea 50°C**	**Cold water 8°C**
**1**	4	8	20	35 excl	20	45 excl
**2**	8	12	16	16	45 excl	25
**3**	4	12	16	16	16	4
**4**	8	25 excl	20	20	20	30
**5**	4	4	8	16	25	25
**6**	8	12	20	20	16	25
**7**	8	12	16	16	20	20
**8**	4	4	16	16	16	16
**9**	4	4	16	16	16	16
**10**	8	12	12	20	20	16
**11**	4	4	16	20	16	16
**12**	4	12	25	25	20	30
**Mean ± S.D. (min) n = 12**	**5.7 ± 2.1**	**10.1 ± 6.0**	**16.8 ± 4.3**	**19.7 ± 5.6**	**20.8 ± 8.1**	**22.3 ± 10.2**
**Mean ± S.D. (min) n = 11 for gelatine and n = 10 for HPMC**	**5.5 ± 2.0**	**8.7 ± 3.9**	**16.5 ± 4.4**	**18.5 ± 3.0**	**18.5 ± 3.0**	**19.8 ± 8.0**

**Potential esophageal retention/"lazy stomach" - visible in profile of capsule**
**Potential "lazy stomach"/transient gastroparesis - delay in gastric emptying visible in the profiles obtained from the capsule and the tablet**

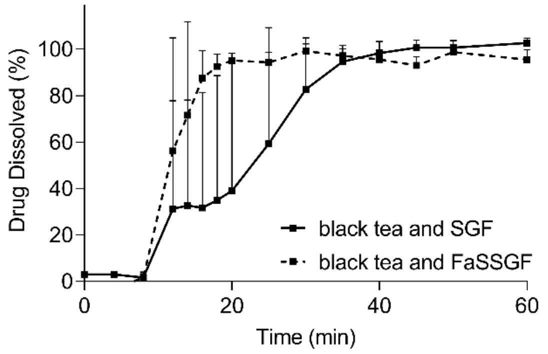

Figure S1: *Comparison of the caffeine release from HPMC capsules in warm black tea with SGF and FaSSGF, n = 3 for both experiments, mean +/- SD.*

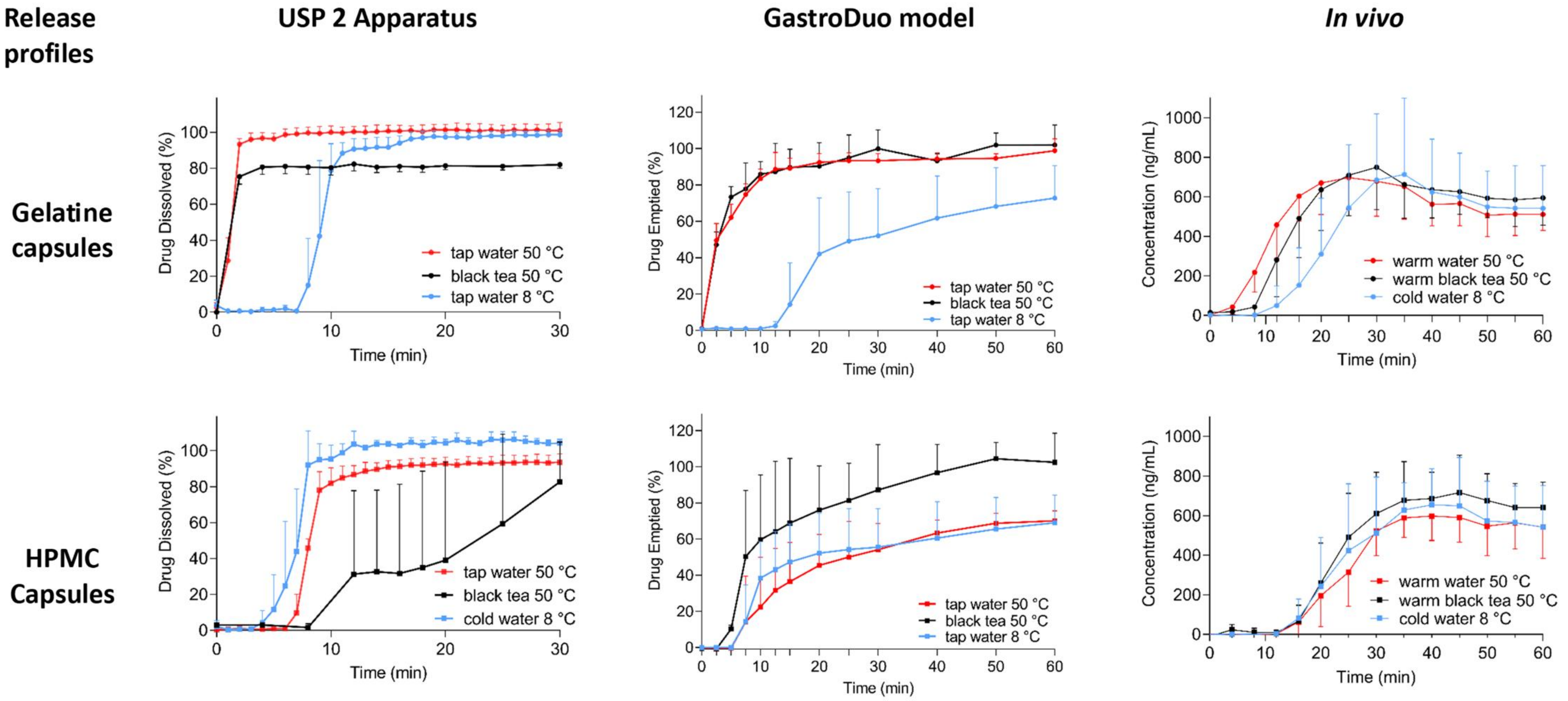

Figure S2: *Comparison of the caffeine profiles in vitro and in vivo released from gelatine and HPMC capsules in different fluids. In USP 2 apparatus, for gelatine capsules n = 3 for all fluids, except warm tap water, where n = 5. For GastroDuo data n = 3 for every fluid, except cold tap water, where n = 4, for in vivo data n = 11 for gelatin capsules in every fluid and n = 10 for HPMC capsules in every fluid. On the graph, mean value +/- SD is presented.*

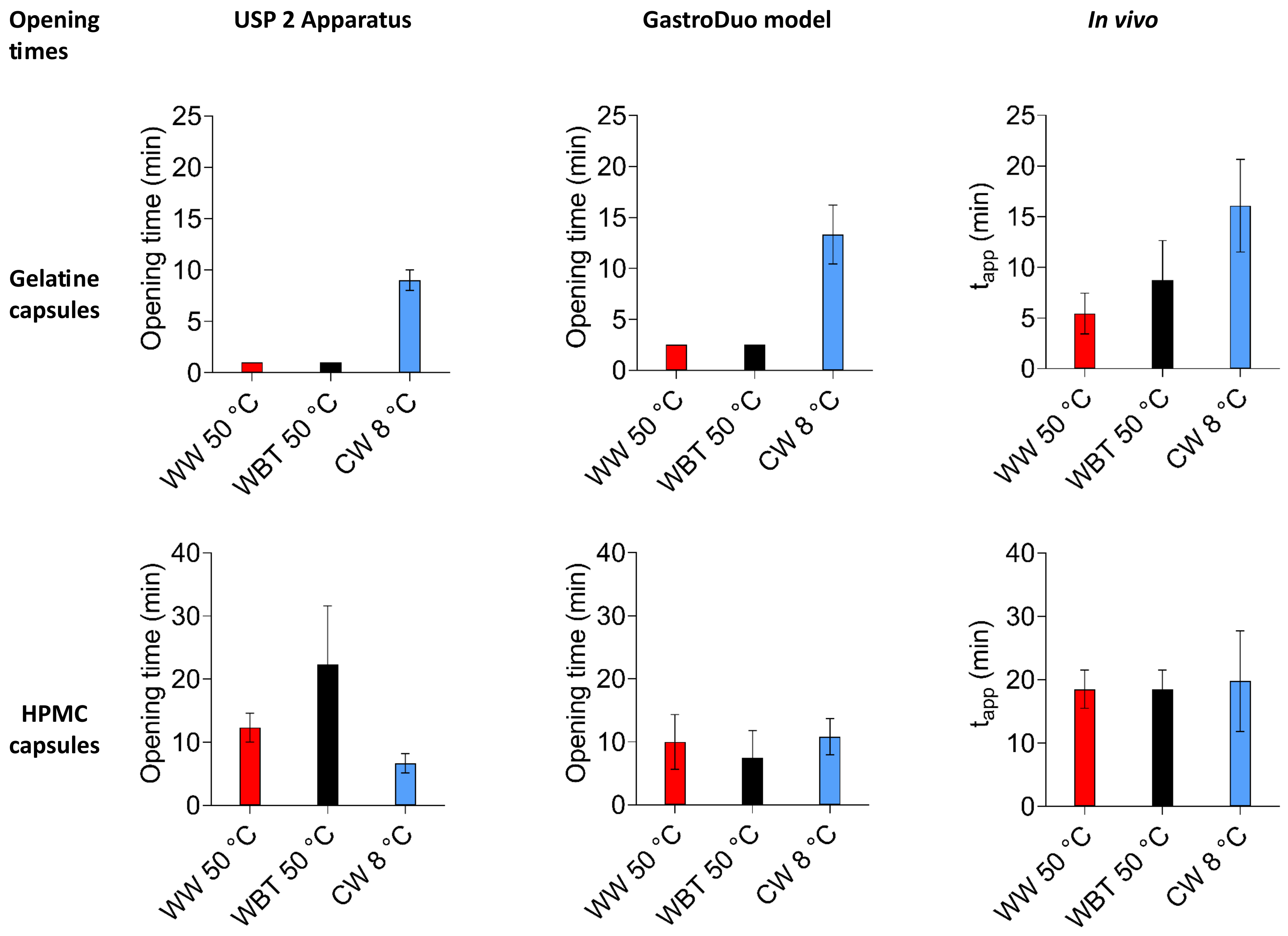

Figure S3: *Comparison of in vitro opening times and in vivo appearance time in Gelatin capsules and HPMC capsules in different fluids WW warm water, WBT warm black tea, CW cold water. In USP 2 apparatus, for gelatine capsules n = 3 for all fluids, except warm tap water, where n = 5. For GastroDuo data n = 3 for every fluid, except cold tap water, where n = 4, for in vivo data n = 11 for gelatin capsules in every fluid and n = 10 for HPMC capsules in every fluid. On the graph, mean value ± SD is presented.*

Figure S4: Individual data from all study participants

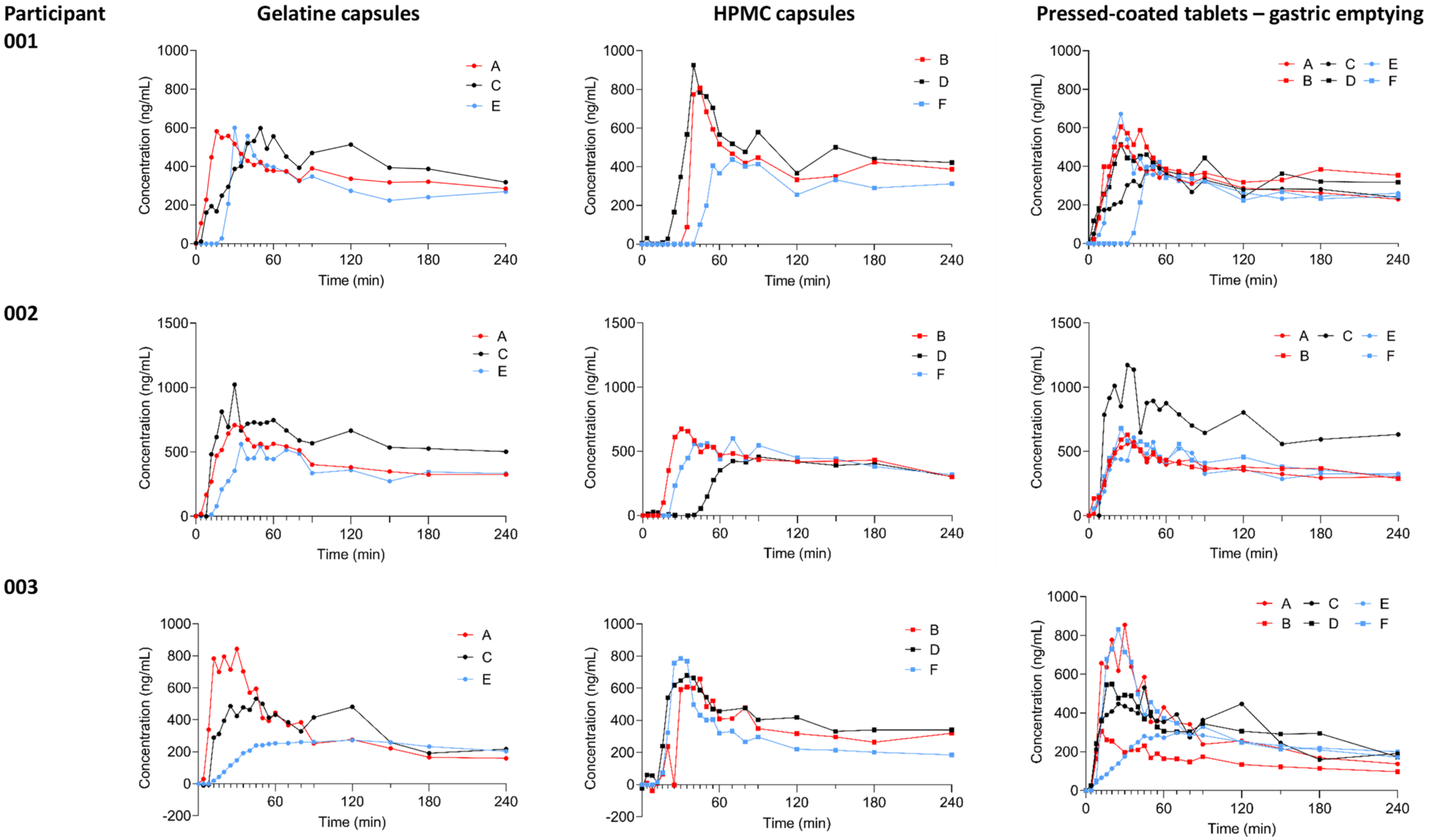

warm water 50 °C red, study arms AB, warm black tea 50 °C black, study arms CD, cold water 8 °C blue, study arms EF

Figure S4: Individual data from all study participants

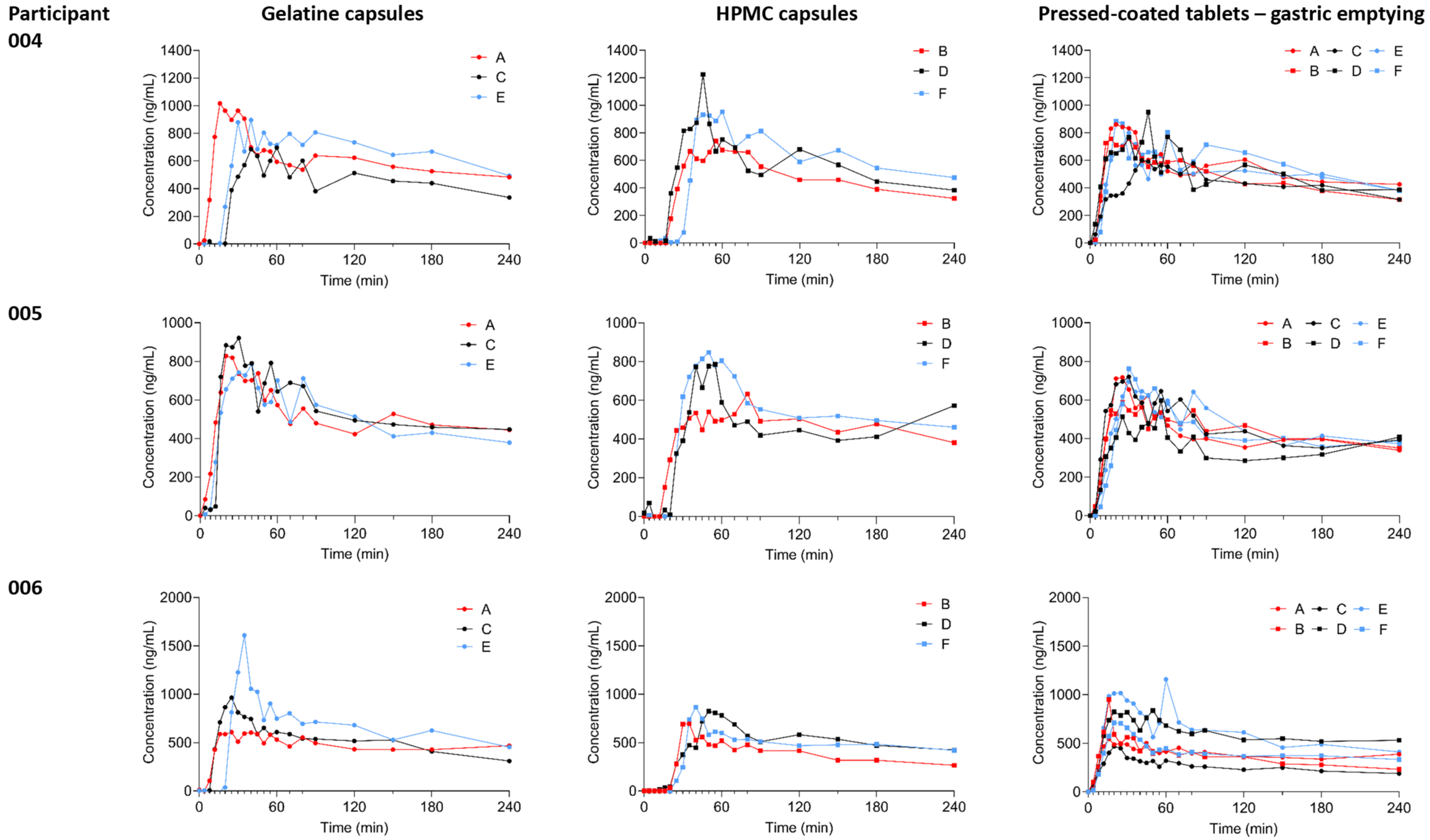

warm water 50 °C red, study arms AB, **warm black tea 50 °C black, study arms CD**, **cold water 8 °C blue, study arms EF**

Figure S4: Individual data from all study participants

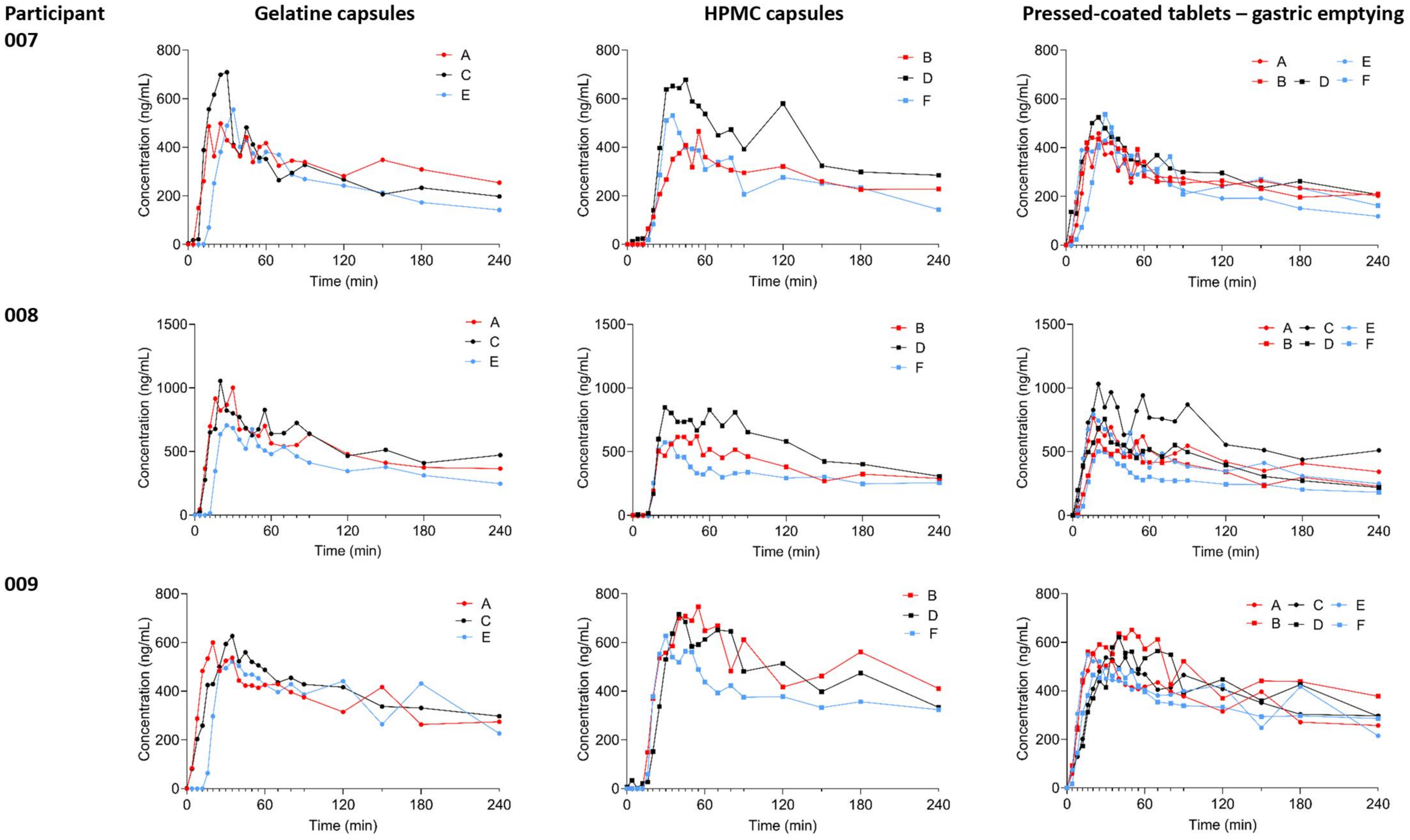

warm water 50 °C red, study arms AB, warm black tea 50 °C black, study arms CD, cold water 8 °C blue, study arms EF

Figure S4: Individual data from all study participants

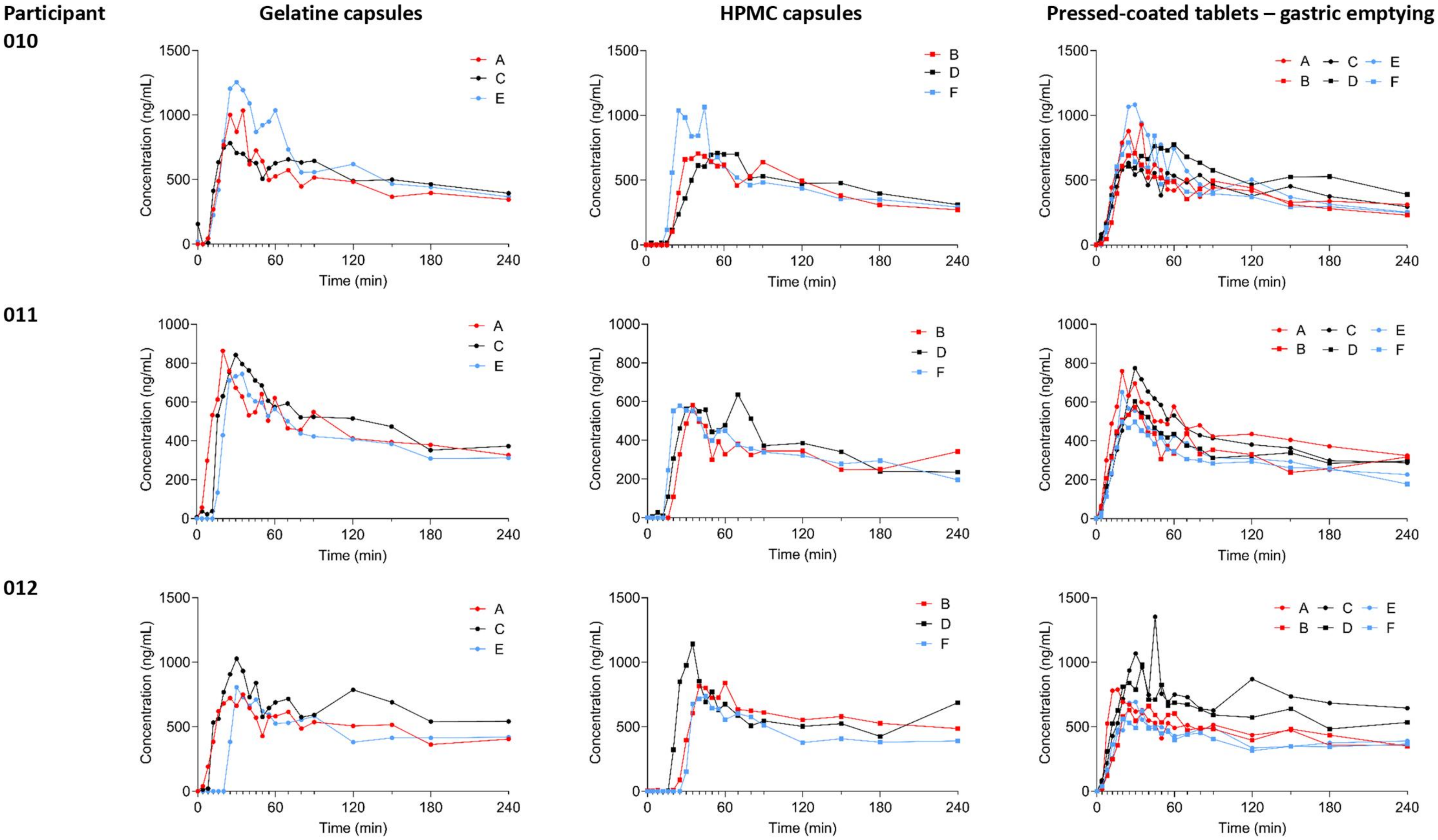

warm water 50 °C red, study arms AB, warm black tea 50 °C black, study arms CD, cold water 8 °C blue, study arms EF

Figure S5: Determination of the correction factor - chromatograms

Natural Caffeine: 10000 ng/mL + Caffeine-C13: 25 ng/mL // Exemplary chromatograms

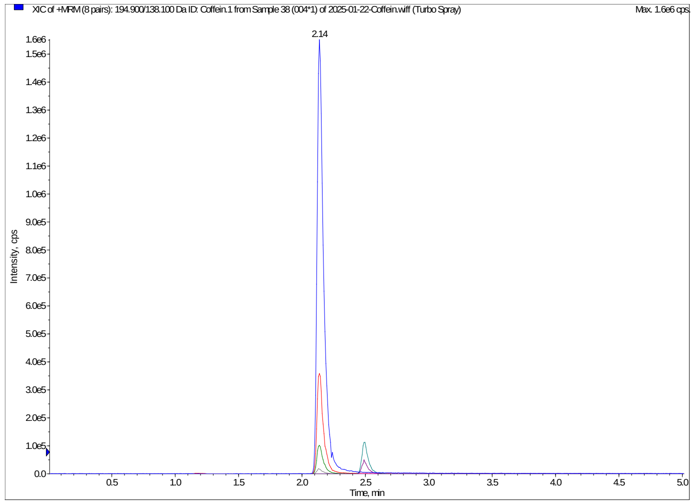

Whole chromatogramm

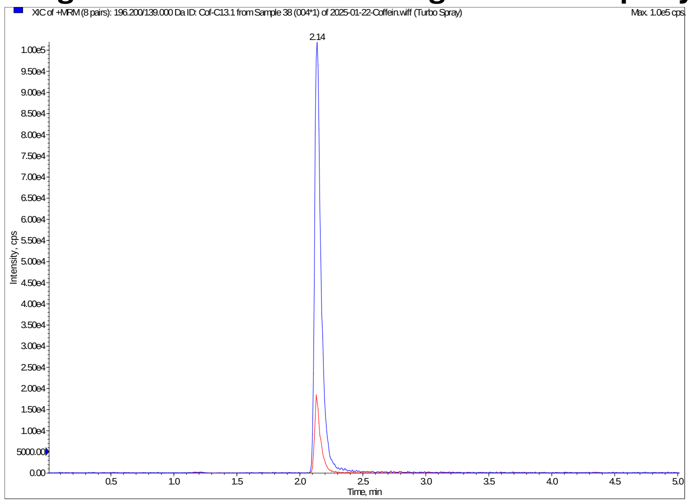

13C1 Caffeine

Caffeine	Caffeine.1	194.9/138.1
	Caffeine.2	194.9/110.0
Caffeine- C13	Caf-C13.1	196.2/139.0
	Caf-C13.2	196.2/111.0
Caffeine-(C13)3	Caf-(C13)3.1	198.2/140.0
	Caf-(C13)3.2	198.2/112.0
8-Chlortheophyllin	8-CT.1	215.1/158.1
	8-CT.1	215.1/130.1

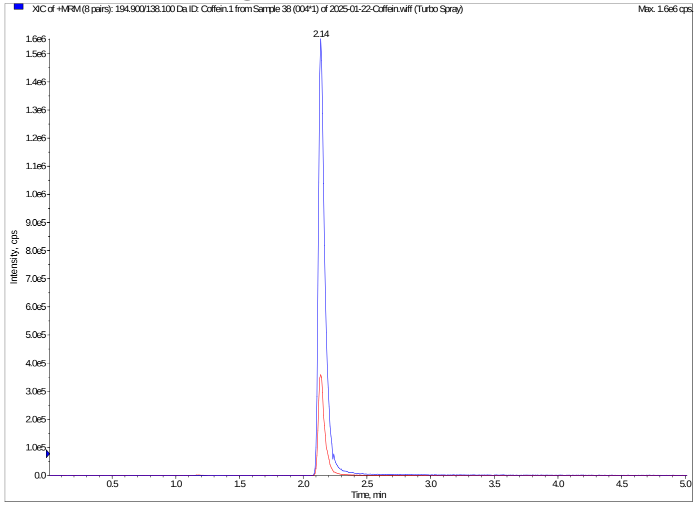

12C Caffeine

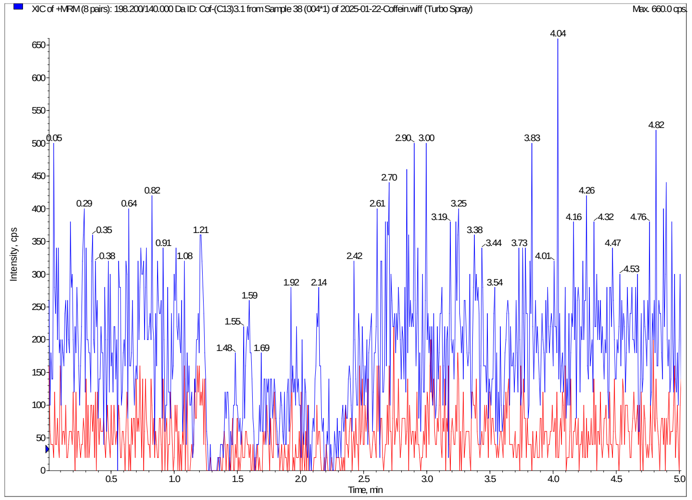

13C3 Caffeine

Measured Caffeine-C13 (ng/mL) [aim: 25 ng/mL]	Measured Caffeine-C12 (ng/mL)	Error in % of caffeine-12C
713	10400	6.62
692	10100	6.60
697	10200	6.59

$$quantification\ error\ (\%) = \frac{measured\ c\ (^{13}C\ caffeine) - actual\ c\ (^{13}C\ caffeine)}{measured\ c\ (^{12}C\ caffeine)} \times 100$$

Figure S5: Determination of the correction factor - chromatograms

Natural Caffeine: 10000 ng/mL + Caffeine-C13: 500 ng/mL // Exemplary chromatograms

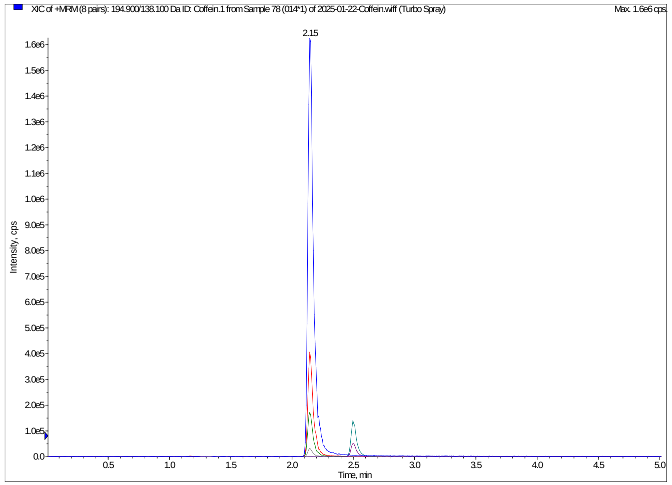

Whole chromatogramm

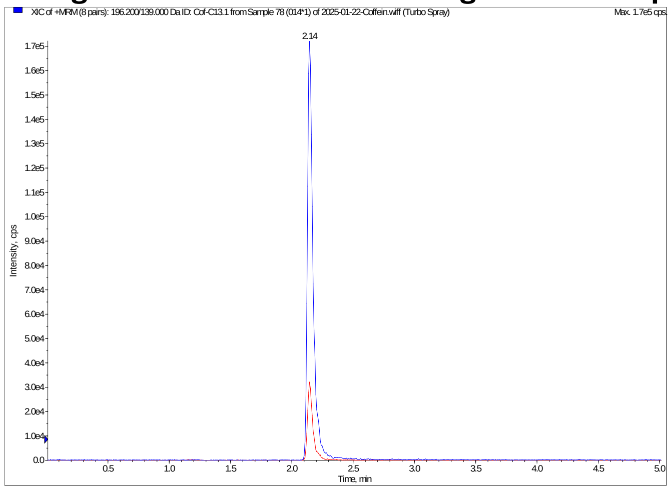

13C1 Caffeine

Caffeine	Caffeine.1	194.9/138.1
	Caffeine.2	194.9/110.0
Caffeine- C13	Caf-C13.1	196.2/139.0
	Caf-C13.2	196.2/111.0
Caffeine-(C13)3	Caf-(C13)3.1	198.2/140.0
	Caf-(C13)3.2	198.2/112.0
8-Chlortheophyllin	8-CT.1	215.1/158.1
	8-CT.1	215.1/130.1

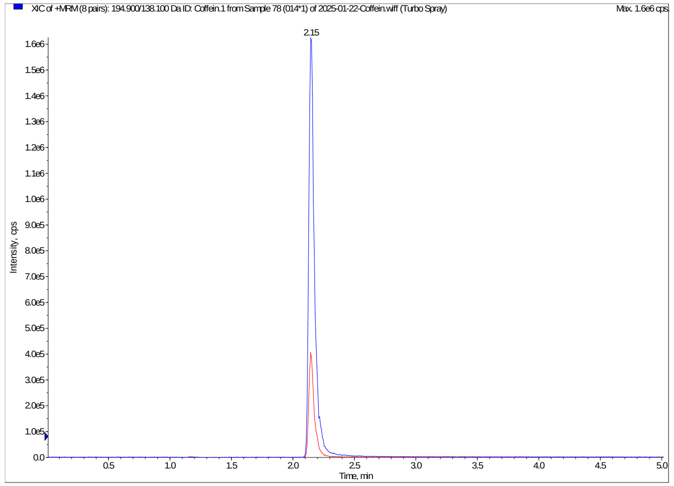

12C Caffeinee

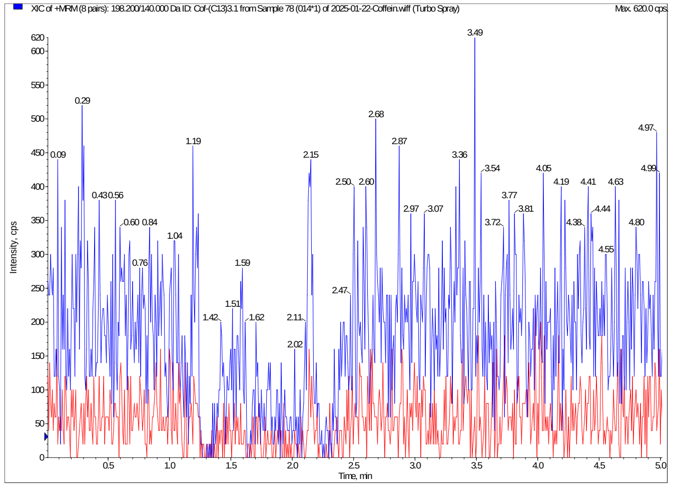

13C3 Caffeine

Measured Caffeine-C13 (ng/mL) [aim: 500 ng/mL]	Measured Caffeine-C12 (ng/mL)	Error in % of caffeine-12C
1060	8820	6.35
1120	9140	6.78
1080	8820	6.58

$$quantification\ error\ (\%) = \frac{measured\ c\ (^{13}C\ caffeine\ ) - \ actual\ c\ (^{13}C\ caffeine)}{measured\ c\ \left(^{12}C\ caffeine\right)} \times 100$$
